# Pencil-Drawn Electrode within Additively Manufactured Devices for Uric Acid Detection

**DOI:** 10.1021/acsomega.6c02346

**Published:** 2026-06-01

**Authors:** Mariana C. Marra, Marina Di-Oliveira, Amanda B. Nascimento, Raquel G. Rocha, Natália C. de Moraes, Eduardo M. Richter, Bruno G. Lucca, Rodrigo A. A. Muñoz

**Affiliations:** † Chemistry Institute, 28119 Federal University of Uberlândia, 38400-902 Uberlândia, Minas Gerais, Brazil; ‡ Department of Chemistry, University of Warwick, CV4 7AL Coventry, U.K.; § Chemistry Institute, Federal University of Mato Grosso do Sul, 79074-460 Campo Grande, Mato Grosso do Sul, Brazil

## Abstract

Disposable electrochemical sensors have attracted increasing attention as low-cost, portable, and environmentally friendly alternatives to conventional analytical methods. Pencil-drawn electrodes (PDEs) offer a simple and sustainable approach, allowing rapid fabrication without conductive inks, sophisticated equipment, or laborious procedures. Here, a PDE was prepared on a stereolithography-additive manufactured substrate assembled on a fused-filament fabrication (FFF) additively manufactured electrochemical cell applied for uric acid (UA) sensing. The electrode was characterized by SEM and Raman spectroscopy, confirming its surface morphology, conductivity, and electrochemical activity. The sensor can be produced at a very low cost using nontoxic and readily available materials, making it suitable for point-of-care applications. The sensor demonstrated good reproducibility, stability, and selectivity, and exhibited electrochemical performance comparable to glassy-carbon electrodes (GCE) and carbon screen-printed electrodes (C-SPE) without any surface pretreatment. The analytical performance of the PDE was evaluated for UA, showing a linear response in the concentration range of 5.0–40.0 μmol L^–1^, with a detection limit (LOD) of 0.2 μmol L^–1^. Finally, it was successfully applied to the determination of UA in synthetic samples. Overall, this study presents a low-cost, eco-friendly, and reliable platform, highlighting the potential of PDEs on additively manufactured platforms for sustainable and accessible analytical chemistry.

## Graphical abstract

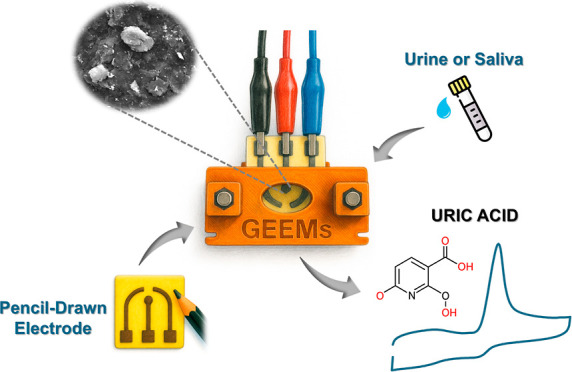

## Introduction

In recent years, the need for affordable and cost-effective analytical tools has increased significantly, especially in healthcare applications. Conventional diagnostic methods often depend on complex instrumentation, specialized personnel, and controlled laboratory environments, which limit their accessibility and increase their costs. This scenario highlights the need for alternative approaches that enable rapid, low-cost, and decentralized analyses.^1,2^

Uric acid (UA) is of particular interest, as it is a key biomarker associated with metabolic disorders such as gout, hyperuricemia, and kidney diseases.^3^ Conventional methods for UA detection, such as chromatography techniques, often require expensive equipment and labor-intensive sample preparation steps.^4,5^

In contrast, electrochemical devices have emerged as a promising solution due to some properties, including ease of portability, high sensitivity, and compatibility with miniaturized systems.^2,6,7^ Furthermore, the miniaturization of electrochemical devices, facilitated by advances in materials and fabrication technologies, has allowed the alternative to bulky and expensive setups with compact, easy-to-use, and battery-powered systems. These aspects make them suitable for point-of-care (POC) analysis, where simplicity, affordability, and rapid response are important.^1,6^

The World Health Organization (WHO) has emphasized the importance of POC diagnostics and proposed the ASSURED criteria (affordable, sensitive, specific, user-friendly, rapid and robust, equipment-free, and deliverable) to guide the development of effective testing platforms.^8,9^ The implementation of these criteria has resulted in the design of miniaturized electrochemical platforms capable of performing reliable detection of different biological analytes, including metabolites, drugs, proteins, and nucleic acids, using minimal sample volumes from complex matrices such as blood, saliva, or urine.^1,6^

In recent years, additive manufacturing (3D printing) technology has been increasingly employed as a versatile tool for fabricating a wide range of devices in analytical chemistry.^10–13^ In this approach, three-dimensional (3D) models are designed using computer-aided design (CAD) software in geometries tailored to specific applications and subsequently manufactured through layer-by-layer additive printing. Compared to traditional subtractive manufacturing technologies, 3D-printing is a fast, cost-effective approach with minimal waste generation.^14,15^

In the field of electrochemistry, 3D printing enables the direct fabrication of tailored electrochemical cells, electrode designs, and integrated sensing platforms with complex geometries that are difficult to achieve through conventional manufacturing.^14,15^ The ability to generate specific designs rapidly and at low cost significantly improves prototyping and device optimization. By reducing dependence on specialized machining and microfabrication facilities, 3D printing is fostering the development of portable electrochemical devices, supporting the growing demand for decentralized analytical technologies.^14^

Within this context, pencil-drawn electrodes (PDEs) have attracted significant attention as one of the most accessible and sustainable fabrication methods, since they do not require conductive inks, complex equipment, or laborious procedures.^16^ This approach relies on the simple mechanical abrasion of a graphite pencil onto a solid substrate, creating conductive carbon tracks with customizable dimensions and geometries.^17^

Although cellulose-based material has been the most popular substrate for PDEs because of its availability and biodegradability, its porous and hydrophilic nature often results in poor mechanical integrity and signal instability during electrochemical measurements.^17^ Other substrates, such as polyvinyl chloride (PVC), polyester among other materials, have been explored to overcome these drawbacks;^16,18–21^ however, these substrates also have specific limitations in terms of reproducibility, stability, or sustainability. Thus, the research for new and optimized substrates remains an active field, aiming to improve electrode performance while maintaining a straightforward, low-cost, and eco-friendly fabrication process.

Among these alternatives, stereolithography-based additively manufactured substrates using sustainable resins have been previously proposed for pencil-drawn electrodes by de Moraes et al., addressing many limitations of conventional substrates.^22^ Their intrinsically hydrophobic surface prevents solution leakage, eliminating the need for additional sealing steps or surface treatments to improve graphite adhesion or mitigate exfoliation, thus ensuring a stable and reliable platform for electrochemical measurements. Moreover, the use of biodegradable photosensitive resins combined with nontoxic graphite pencils enables the fabrication of environmentally friendly and low-cost PDEs. Although this platform has demonstrated promising performance, its broader analytical applicability and the influence of key fabrication parameters remain insufficiently explored. In recent years, PDEs have been successfully applied to the detection of a wide range of analytes, including heavy metals,^23^ pesticides,^24^ and pharmaceutical compounds.^25^

Therefore, this work proposes a sustainable electrochemical platform for UA determination employing pencil-drawn electrodes on an additively manufactured device. The proposed sensor can be fabricated rapidly and cost-effectively without requiring any pre- or post-treatment steps, thus fully adhering to the principles of Green and Sustainable Analytical Chemistry. Its analytical performance for UA detection was comprehensively evaluated, demonstrating high potential for point-of-care testing and deployment in resource-limited environments.

## Experimental Section

### Standards and Solutions

All aqueous solutions were prepared using deionized water with a resistivity of at least 18 MΩ cm (Millipore Direct-Q3 water purification, MA, USA). Urea (≥99% wt.) and uric acid (≥99% wt.) were obtained from Sigma-Aldrich (St. Louis, USA). Potassium ferricyanide (≥99% wt.), sodium nitrite (≥98% wt.), sodium chloride (≥99% wt.), monopotassium phosphate (≥98% wt.), and sodium hydrogen carbonate (≥99.5% wt.) were acquired from Labsynth (Diadema, Brazil). Potassium chloride (≥99.5% wt.) was purchased from Êxodo Científica (São Paulo, Brazil). Sodium phosphate dibasic (≥99% wt.), sodium sulfate (≥99% wt.), potassium thiocyanate (≥99% wt.), acetic acid (≥99% w/v), and phosphoric acid (≥85% w/v) were obtained from Vetec (Rio de Janeiro, Brazil). Boric acid (≥99% wt.) was obtained from AppliChem Panreac (Barcelona, Spain). Sodium hydroxide (≥98% wt.) and ethanol (≥95% w/v) were obtained from ChemiFlex (São Bernardo do Campo, Brazil). All reagents were of analytical grade and used as received without further purification.

A mixture of phosphoric, acetic, and boric acids (each at 0.04 mol L^–1^) was used to prepare Britton–Robinson (BR) buffer solutions. The pH of the BR solutions was adjusted with a 1 mol L^–1^ NaOH, which was employed as the supporting electrolyte before use.

Synthetic saliva was prepared following a modified literature procedure,^26^ by mixing 0.33 g L^–1^ potassium thiocyanate, 0.26 g L^–1^ sodium phosphate dibasic, 1.30 g L^–1^ urea, 0.70 g L^–1^ sodium chloride, and 1.20 gL^–1^ potassium chloride. Synthetic urine was prepared as described in the literature,^27^ using 0.37 g L^–1^ ascorbic acid, 0.04 g L^–1^ calcium chloride, 2.10 g L^–1^ sodium hydrogen carbonate, 0.07 g L^–1^ uric acid, 9.99 g L^–1^ urea, 0.40 g L^–1^ citric acid, 1.41 g L^–1^ sodium sulfate, 5.20 g L^–1^ sodium chloride, 0.95 g L^–1^ magnesium sulfate, 0.95 g L^–1^ monopotassium phosphate, and 1.30 g L^–1^ ammonium chloride. Both synthetic saliva and synthetic urine samples were spiked with UA at one concentration level and analyzed after simple dilution in supporting electrolyte.

### Sensor and Electrochemical Cell Production

Drawing pencils from Faber-Castell (6B grade, Faber-Castell Brazil, São Carlos, SP, Brazil) and Cis (6B grade, Hindustan Pencils Pvt. Ltd., Mumbai, India) were acquired and tested for electrode fabrication. A customized additively manufactured (AM) electrochemical cell was constructed, according to de Moraes and co-workers.^22^ Briefly, this cell consists of a PLA body fabricated by FFF technique with cover and base connected by nut knobs. The substrate at which the PDE is fabricated was produced by stereolithography AM and sealed with an O-ring (ø = 20 mm) to prevent leakage. The internal reservoir has a maximum volume of around 1.7 mL, although smaller volumes can also be used.

To fabricate the stereolithography AM substrate, a biodegradable photosensitive resin for 3D printing (Line Iron 70/30, skin color, 405 nm), purchased from Quanton 3D (Belo Horizonte, MG, Brazil), was used. After printing the substrate in the desired geometry (30 mm width × 35 mm height × 1 mm thickness), it was initially cleaned with ethanol. Then, the adhesive mask was applied to the rougher side of the substrate, and the three-electrode configuration (working electrode (WE, ø = 4 mm), counter electrode (CE), and pseudocarbon reference electrode (RE)) was drawn using pencils from the selected brands. During this step, a multimeter was employed to monitor the electrical resistance at the electrode to ensure good reproducibility between devices, with target resistances of approximately 300 Ω. Subsequently, the adhesive mask was removed, and the PDE was positioned in the custom AM electrochemical cell for measurements, with electrical connections established directly to the electrode contacts using alligator clips (Figure 1).

**Figure 1:**
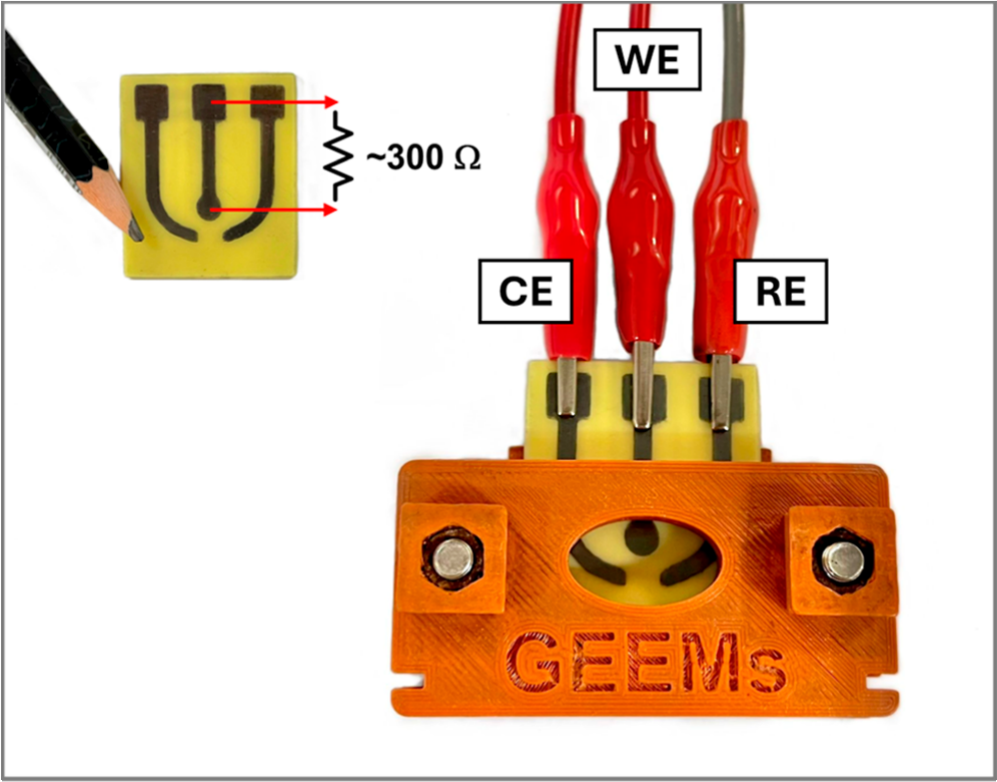
PDE assembled on the custom AM electrochemical cell, illustrating the electrical connections to the working (WE), counter (CE), and reference (RE) electrodes, as employed during the experiments. The electrode layout and the measured resistance (approximately 300 Ω) are also shown in the figure (right side).

### Electrochemical Measurements

All electrochemical measurements were conducted using a PGSTAT204N potentiostat/galvanostat (Metrohm Autolab BV, Utrecht, The Netherlands). Cyclic voltammetry (CV) of the redox probe [Fe­(CN)_6_]^3–/4–^ (2.0 mmol L^–1^ in 0.1 mol L^–1^ KCl solution) at 50 mV s^–1^ was employed for sensor optimization and characterization, while differential pulse voltammetry (DPV) was employed for UA quantification, which was carried out in BR buffer (pH 2), as defined from the pH optimization study, since this buffer enables systematic pH variation while maintaining a constant electrolyte composition. UA concentrations in synthetic samples were determined using the standard addition method. Data acquisition and processing, including baseline correction of DPV scans, were performed using NOVA 2.1.7 software.

Electrochemical impedance spectroscopy (EIS) measurements were carried out at the half-wave potential (+0.02 V *vs* pseudocarbon (PDE), +0.08 V *vs* pseudocarbon (GCE), or +0.22 V *vs* Ag pseudoreference (C-SPE)) in the presence of 2.0 mmol L^–1^ [Fe­(CN)_6_]^3–/4–^ in 0.5 mol L^–1^ KCl, applying an alternating potential with an amplitude of 10 mV over a frequency range from 50 kHz to 0.1 Hz. The experimental data were fitted using the Randles equivalent circuit to determine the charge-transfer resistance (*R*_ct_) associated with the [Fe­(CN)_6_]^3–/4–^ redox system for each electrode.

The double-layer capacitance (*C*_dl_) was determined from CV measurements performed between 0.0 and +0.30 V (vs pseudocarbon) at various scan rates in 0.1 mol L^–1^ KCl, following literature protocols.^26^ A plot of the difference between the anodic and cathodic currents at +0.15 V, normalized by the geometric electrode area (A = 0.15 cm^2^), versus the scan rate (5–30 mV s^–1^) was constructed. The *C*_dl_ was obtained from the slope of the resulting linear fit.

### Surface Characterization

Raman spectra were recorded using a LabRAM HR Evolution spectrometer (HORIBA, Japan) equipped with an OSD Syncerity detector and a 532 nm excitation laser. Scanning electron microscopy (SEM) images were acquired on a Tescan VEGA 3 LMU microscope operated at 20 kV.

Next, atomic force microscopy (AFM) characterization was performed using a scanning probe microscope (SPM-9600, Shimadzu, Japan) operated in dynamic force mode. Silicon cantilevers (PPP-NCHR, Nanosensors, Switzerland) were employed, with a nominal resonance frequency of 330 kHz and a spring constant of 42 N m^–1^, and typical dimensions of 125 μm in length, 30 μm in width, and 4 μm in thickness. Images were acquired over a 30 μm × 30 μm scan area with a vertical range of 5 μm.

Contact angle measurements of each electrode surface were carried out using a smartphone mounted on a universal holder. Images were captured 10 s after deposition of a deionized water droplet onto the working electrode surface, and the contact angle was calculated using GeoGebra software.^28^

## Results and Discussion

### Optimization and Characterization

The electrochemical performance of electrodes prepared from two graphite pencil brands was evaluated following their deposition onto the AM substrate. For both cases, identical fabrication parameters were employed, with the conductive tracks generated by applying 30 sequential strokes to ensure consistent graphite coverage. The selection of the most suitable pencil was based on the cyclic voltammetric response obtained in 2.0 mmol L^–1^ [Fe­(CN)_6_]^3–/4–^ containing 0.1 mol L^–1^ KCl at 50 mV s^–1^ (Figure 2A). The resulting CVs exhibited well-defined and reversible redox peaks, indicating that the continuous graphite traces provided adequate electrical conductivity for sensor operation. These results also demonstrate that the AM substrate possesses sufficient surface roughness to promote effective graphite deposition, thereby eliminating the need for additional pretreatments and enabling a rapid and cost-efficient electrode fabrication process.

**Figure 2:**
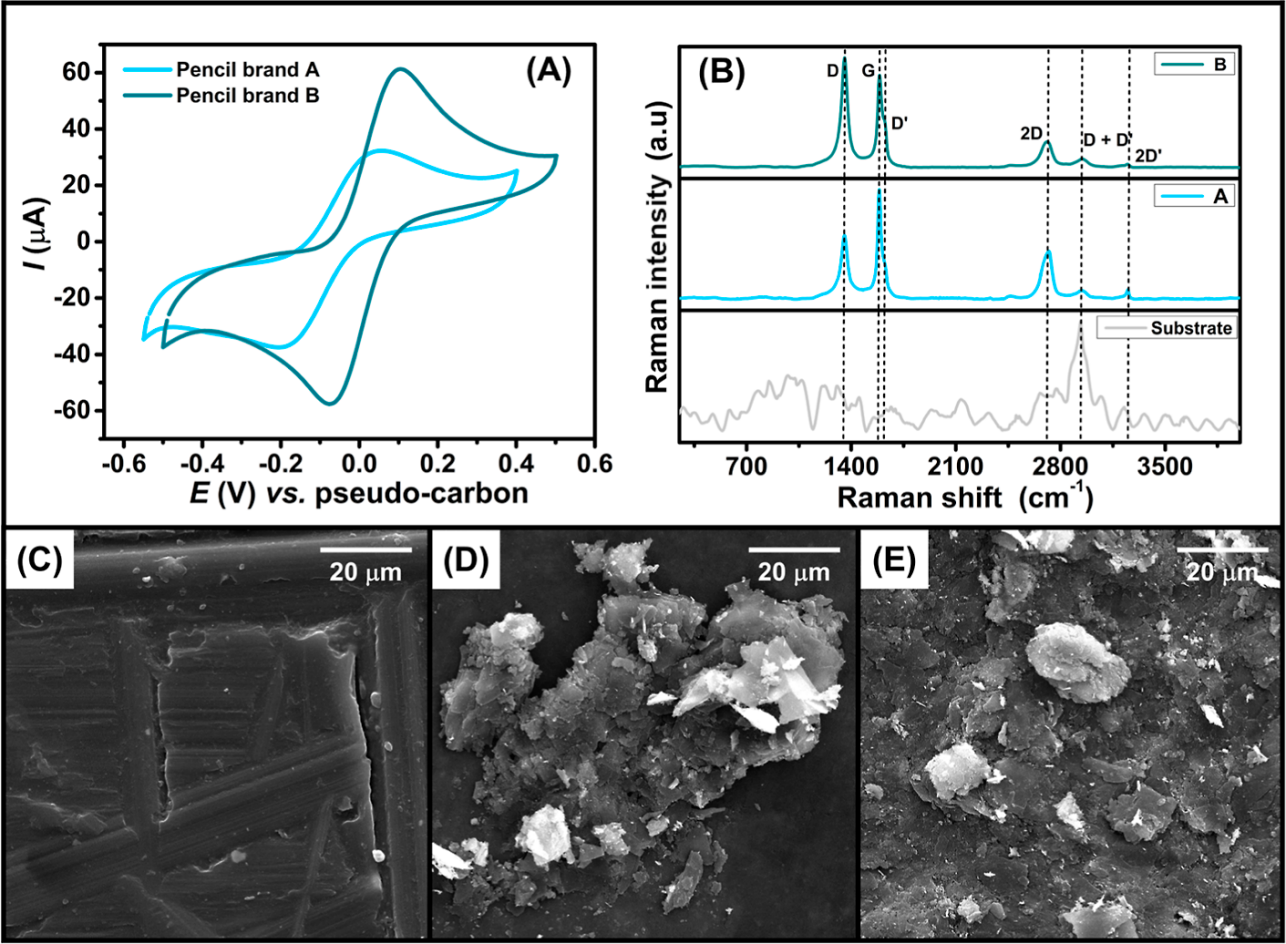
(A) CV obtained with electrodes fabricated using different brands of graphite pencils, recorded in 2 mmol L^–1^ [Fe­(CN)_6_]^3–/4–^ containing 0.1 mol L^–1^ KCl at a scan rate of 50 mV s^–1^. (B) Raman spectra of the substrate (gray line), pencil brand A (light blue line), and pencil brand B (dark blue line). Scanning electron microscopy (SEM) images of (C) the substrate, (D) pencil brand A, and (E) pencil brand B.

As shown in Figure 2A, electrodes produced with pencils from brand B demonstrate superior electrochemical responses, exhibiting a higher anodic peak current (∼48 μA) and a smaller peak-to-peak separation (Δ*E*_p_ ∼ 174 mV) compared to electrodes fabricated with brand A, which presented lower peak currents (∼24 μA) and a significantly larger Δ*E*_p_ (∼242 mV). These results suggest that pencil B provides a more homogeneous and continuous deposition of graphite onto the substrate, contributing to the enhanced electrochemical response.

To explore the relationship between these electrochemical behaviors and the morphological features of the electrodes, SEM images of PDEs obtained from different pencils were analyzed (Figure 2D,E). As observed, the substrate displayed a continuous texture across the images, as previously reported for this type of material.^29^ Additionally, the substrate (Figure 2C) showed slight roughness and surface defects, which can be attributed to microirregularities on the 3D-printer build plate. This roughness likely played an important role in facilitating the deposition of graphite layers during the electrode fabrication process.

After graphite deposition, it is possible to observe that the substrate was completely covered, forming a continuous graphite layer. Notably, the electrodes produced with pencil brand B appear to exhibit a thicker and more uniform coating, which likely contributes to the enhanced electrochemical response observed in Figure 2A.

Raman spectra (Figure 2B) provide complementary structural insights into graphitic-based materials. The spectra acquired for both evaluated pencil brands exhibit the characteristic carbonaceous bands, namely the D (1355 cm^–1^), G (1587 cm^–1^), D′ (1618 cm^–1^), 2D (2722 cm^–1^), D + D′ (2937 cm^–1^), and 2D′ (3238 cm^–1^) bands.^30^ In contrast, the spectrum recorded for the bare substrate shows no defined bands, only noise, as expected. The D band is associated with structural defects and disorder within the graphite layers, whereas the G band corresponds to the in-plane vibrational mode of sp^2^-hybridized carbon atoms in the graphitic lattice. The D′ band, typically deconvoluted from the G band, further indicates the presence of structural defects in graphitic materials.^31^ The 2D band provides valuable information regarding the number of stacked graphene layers, while the D + D′ and 2D′ bands arise from higher-order combination modes.^30^ Overall, the relative intensities and positions of these Raman features offer essential insights into the structural characteristics and physicochemical properties of graphitic materials.

Electrodes fabricated using the brand B pencil exhibited a D band of higher intensity than the G band, yielding an *I*_D_/*I*_G_ ratio of 1.25. This value indicates a high density of structural defects and edge sites in the graphite material.^31,32^ Moreover, the Raman spectrum obtained from the brand B electrode displayed an *I*_2D_/*I*_G_ ratio of 0.29, which is characteristic of multilayer graphene structures.^33,34^ The ratio between the areas of the D and D′ bands provide additional insights into the nature of defects within the graphitic lattice. For this electrode, an *A*_D_/*A*_D′_ ratio of 7.99 was obtained, suggesting the coexistence of edge defects and basal-plane defects.^35^

In contrast, electrodes fabricated using the brand A pencil exhibited deconvoluted spectra in which the D band was less intense than the G band, yielding an *I*_D_/*I*_G_ ratio of 0.59. This result suggests a surface with fewer structural defects compared with the electrode produced using brand B. Moreover, the Raman spectrum of the brand A electrode displayed an *I*_2D_/*I*_G_ ratio of 0.48, indicative of multilayer graphene with a smaller number of stacked layers than that observed for the brand B electrode. Additionally, an *A*_D_/*A*_D′_ ratio of 4.33 was obtained for this electrode, suggesting that the defects in this material are predominantly edge-type defects.^35^

These findings indicate that electrodes fabricated using pencils from different brands exhibit distinct structural characteristics, implying that the graphite composition of each pencil likely differs, even though both electrodes were produced using the same fabrication procedure. This interpretation is further supported by the observation that brand B leads to a more uniform and continuous graphite layer on the AM substrate, which is consistent with the superior voltammetric performance observed for these electrodes.

Subsequently, AFM imaging was performed to further evaluate the morphological and topographical features of the PDE surface. The analysis was conducted on the bare substrate, the pencil-drawn electrode with a single graphite layer, and the electrode with 30 layers of the redeposited graphite composite film, using brand B pencil, enabling a comparative assessment of surface morphology and homogeneity, Figure S1A–C.

The root-mean-square roughness (RMS) values were 479.1 nm for the bare substrate, 258.9 nm for the PDE with a single graphite layer, and 55.9 nm for the PDE with 30 layers of redeposited graphite. The relatively high roughness observed for the bare substrate is consistent with the characteristics of surfaces produced by 3D-printing and can be attributed to microirregularities inherent to the printing process. Upon deposition of a single graphite layer, a noticeable reduction in surface roughness is observed, which can be attributed to the formation of a conductive film that partially covers and smooths the underlying substrate features. Similar behavior was observed by Srinivas et al., who reported a roughness of approximately 18 nm for bare pencil graphite electrodes.^36^

This effect becomes more pronounced with increasing number of graphite layers. For the electrode with 30 layers, the substantial decrease in roughness suggests a more effective coverage of the substrate, leading to the formation of a more continuous and uniform graphite film. This progressive smoothing of the surface supports the hypothesis that repeated pencil-drawing promotes the development of an increasingly homogeneous conductive network across the substrate.

Despite the overall decrease in surface roughness with increasing number of deposited graphite layers, it is important to note that the surface of the PDE with 30 layers still exhibits distinct topographical features associated with the conductive material itself. In this case, the contribution from the underlying substrate becomes significantly reduced, and the observed surface morphology is predominantly governed by graphite film.

These AFM results agreed with the morphological changes observed in the SEM analysis, further confirming the progressive formation of a more continuous and homogeneous graphite film with an increasing number of deposited layers. At the same time, the presence of surface irregularities associated with the graphite phase suggests the development of an interconnected conductive network containing intrinsic defects. Such structural features are consistent with the improved electrochemical performance observed for the PDE.

Given that pencil brand B produced the most favorable electrochemical and morphological results, the subsequent experiments were conducted using this material. In the next step, the number of graphite layers was also evaluated during the electrode fabrication, using pencil brand B. This study was assessed considering the following parameters: resistance (Ω) measured with multimeter, anodic peak current (*I*_pa_), and peak to peak separation (Δ*E*_p_). CVs experiments were recorded using pencil-drawn electrodes with varying numbers of graphite layers (from 1 to 40) in the presence of 2.0 mmol L^–1^ [Fe­(CN)_6_]^3–/4–^ in 0.1 mol L^–1^ KCl at 50 mV s^–1^ (Figure 3A). As the number of graphite layers increased, the electrical resistance progressively decreased and remained nearly constant after approximately 20 layers. This behavior indicates that 20 graphite layers are sufficient to achieve optimal electrical conductivity and a stable voltammetric response (Figure 3B). However, in terms of electrochemical performance, *I*_pa_ increased significantly with the number of layers, reaching a maximum of 54 μA at 30 layers (from 9 μA for a single layer) (Figure 3C). Similarly, Δ*E*_p_ decreased with increasing layers (from ∼1500 to 130 mV), reflecting an apparent improvement in electron transfer kinetics (Figure 3D).

**Figure 3:**
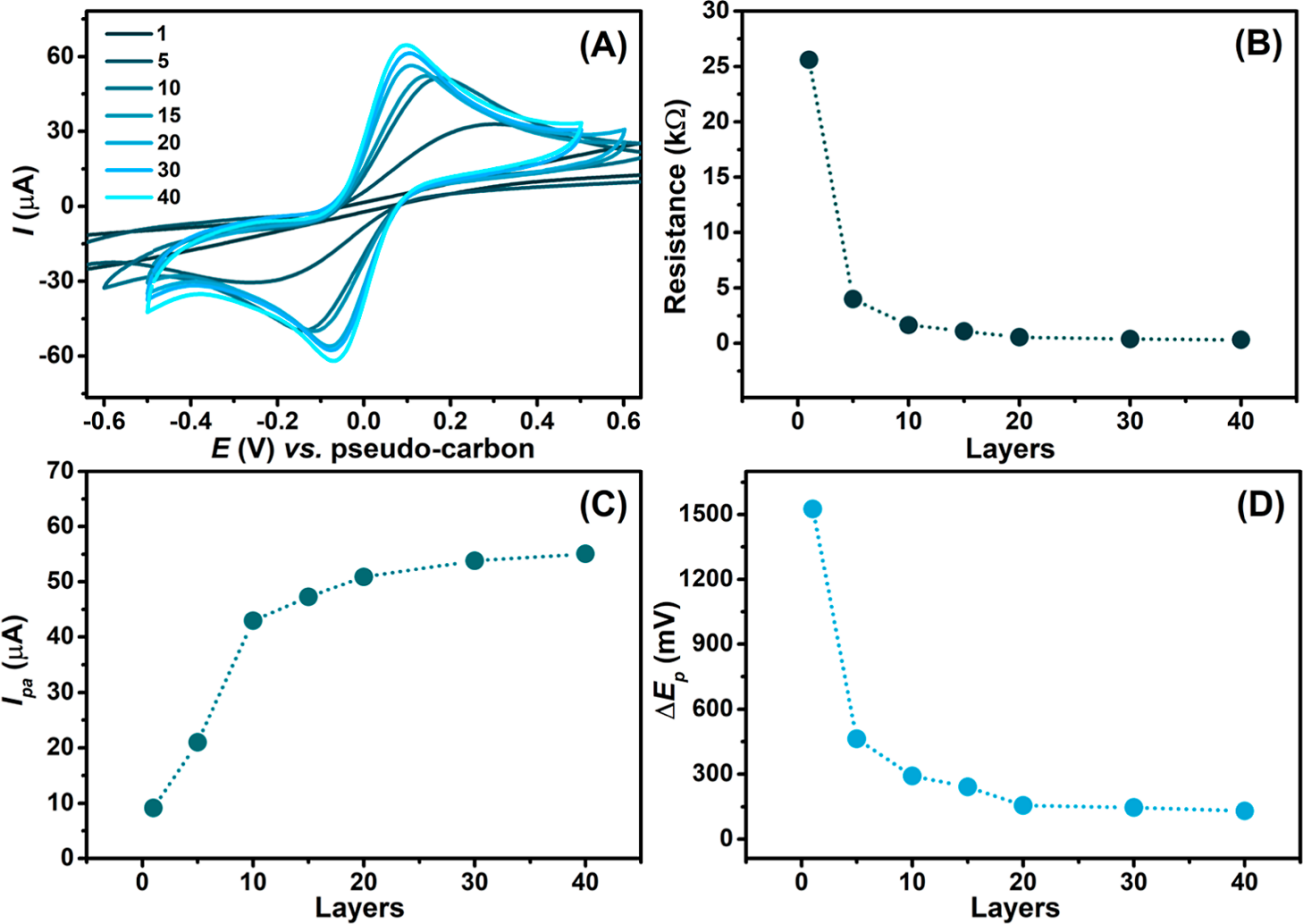
**(A)** Cyclic voltammograms recorded using pencil-drawn electrodes with varying numbers of graphite layers in the presence of 2.0 mmol L^–1^ [Fe­(CN)_6_]^3–/4–^ in 0.1 mol L^–1^ KCl at a scan rate of 50 mV s^–1^. Dependence of **(B)** electrical resistance, **(C)**anodic peak current (*I*_pa_), and **(D)** peak-to-peak separation (Δ*E*_p_) as a function of the number of graphite layers used in electrode fabrication.

This improvement in electron transfer kinetics can be understood because of structural and interfacial changes occurring during the progressive buildup of the graphite composite film throughout the electrode fabrication process, which is based on the redeposition of the same material onto the substrate.^37^ In particular, the simultaneous decrease in electrical resistance, increase in peak current, and reduction in Δ*E*_p_ are consistent with an increase in graphite content and, more importantly, with an enhanced degree of electrical connectivity within the film. This behavior can be interpreted within the framework of percolation theory, which describes the transition from a poorly connected to a fully interconnected conductive network as the density of conductive graphite domains increases.^38^

Percolation theory predicts an S-shaped dependence of electrical conductivity on the fraction of conductive material, comprising three distinct regimes, including insulating, percolation, and fully conductive.^39^ At low numbers of deposited layers of graphite, the system operates in a subpercolation regime, where graphitic domains are spatially separated or only weakly connected,^40^ resulting in high interfacial resistance and inefficient charge transport. Under these conditions, electron transport occurs predominantly through a combination of hopping and tunneling mechanisms, leading to significant ohmic losses and large Δ*E*_p_ values.^39,41^

As additional layers are deposited, the probability of interparticle contact increases, and the system progressively approaches and eventually surpasses the percolation threshold, establishing a continuous conductive network across the electrode surface.^42^

Importantly, although the chemical identity of the material remains unchanged, the percolative transition fundamentally alters the macroscopic electronic properties of the graphite composite film probably through changes in mesostructural organization, particle connectivity, and porosity.^40,43^

In addition to these connectivity effects, Raman spectroscopy (Figure 2B—Brand B) provides evidence for a complementary defect-mediated contribution. The ratio between the areas of the D and D′ bands (*A*_D_/*A*_D′_) was found to be 7.99, which indicates the coexistence of edge-type and basal-plane defects within the graphitic structure.^44^ Notably, for the electrode fabricated with 30 layers, the Raman response confirms a significant population of edge-like defects, which are widely recognized as highly electroactive sites due to their higher density of electronic states and more favorable charge transfer properties compared to basal-plane domains.^45–47^ The presence of these defects further enhances electron transfer at the electrode/electrolyte interface, contributing to the observed decrease in anodic peak potential and Δ*E*_p_.

The reproducibility and repeatability of the electrodes were systematically evaluated in the presence of 2.0 mmol L^–1^ [Fe­(CN)_6_]^3–/4–^ in 0.1 mol L^–1^ KCl (Figure S2). Batch-to-batch reproducibility demonstrated consistent voltammetric profiles across three production batches, with a relative standard deviation (RSD) of 5.0% for *I*_pa_, confirming fabrication reliability (Figure S2A). Analyst-to-analyst reproducibility was assessed using three independent operators, showing minor variations and an overall RSD of 2.5% for *I*_pa_ (Figure S2B). Electrode-to-electrode reproducibility within a single batch was evaluated for five electrodes, yielding an RSD of 3.4% (Figure S2C). In terms of potential, the peak potentials presented relative deviations lower than 2.9% between measurements. Intraelectrode precision was determined from 50 successive voltammograms on a single electrode, providing an RSD of 2.7% for peak current and 7.6% for peak potential (Figure S2D). These results demonstrate that the proposed electrode platform exhibits high reproducibility, repeatability, and measurement reliability.

The temporal stability of the electrode was further evaluated by comparing cyclic voltammograms recorded for the same electrode on day 1 and day 7 (Figure S3). A variation in peak current (Δ*I*_p_ = 20%) and peak potential (Δ*E*_p_ = 30 mV) was observed over this period, which may be attributed to slight surface changes or adsorption of species. These results suggest that, although the electrode maintains a consistent electrochemical response over time, its analytical performance is improved when used shortly after preparation.

After that, contact angle measurements were performed to evaluate the wettability of the substrate before and after graphite deposition. The contact angle primarily depends on the interactions between the chemical groups at the surface and the droplet composition.^48^ The AM substrate exhibited a contact angle of (47.9 ± 2.8)° (Figure S4A), indicating a relatively hydrophilic surface. After deposition of graphite layers, the contact angle slightly increased to (57.4 ± 1.5)° (Figure S4B), reflecting the slightly more hydrophobic nature of the carbon tracks compared to the AM substrate.^49^ This moderate hydrophobicity helps retain the electrolyte solution on the electrode surface, eliminating the need for hydrophobic barriers typically used in paper-based electrodes.

The electrochemical response of the sensors for 2.0 mmol L^–1^ [Fe­(CN)_6_]^3–/4–^ in 0.1 mol L^–1^ KCl was also evaluated using different reference electrodes (RE) (Figure S5). [Fe­(CN)_6_]^3–/4–^ in 0.1 mol L^–1^ KCl was reduced with an *E*_pc_ of −0.078 V using pseudocarbon RE, representing a shift in the cathodic peak potential (*E*_pc_) of ∼0.200 V from the off-chip Ag|AgCl|KCl_(sat.)_ RE. *E*_pc_ slightly changed for pseudocarbon RE and pseudosilver RE, demonstrating that pseudocarbon RE can be used for the proposed system. Considering the time to paint and dry completely, dispensing silver ink might be an attractive feature since it reduces the electrode manufacturing time and cost.

To further investigate the influence of the reference system, CV measurements were carried out using an external reference electrode over 50 consecutive scans (Figure S6). This approach enabled a direct comparison with the results obtained using the pseudoreference electrode (Figure S2D). The variation in peak potential was significantly lower when using the external reference electrode (1.1%) compared to the pseudoreference electrode (7.6%), indicating improved potential stability. Nevertheless, the pseudoreference electrode still provided a consistent electrochemical response, supporting its applicability in the proposed system within shorter times.

It is important to note that the carbon-based pseudoreference electrode used in this work does not provide a well-defined thermodynamic reference potential, and its potential may vary depending on sample composition, ionic strength, and measurement conditions.^50,51^ As a result, absolute potential values may not be directly comparable to those obtained with conventional reference electrodes, and some degree of potential drift may occur, particularly over extended operation times.^52^

Despite these limitations, such electrodes are well suited for short-term measurements, where good repeatability can be achieved under controlled conditions. In this context, the use of a carbon pseudoreference electrode enables a simplified and fully integrated device architecture, avoiding additional materials or conditioning steps, which is advantageous for low-cost and disposable sensing platforms.

Additionally, open circuit potential (OCP) measurements were carried out to assess the potential stability of the pseudoreference electrode (Figure S7). The OCP was monitored using pseudocarbon as the working electrode and Ag|AgCl|KCl_(sat.)_ as the reference electrode in a solution containing 2.0 mmol L^–1^ [Fe­(CN)_6_]^3–/4–^ and 0.1 mol L^–1^ KCl. The relatively stable OCP values observed over time indicate that the pseudoreference electrode provides a consistent potential, corroborating its suitability for the proposed system.

Next, the electrochemical response for [Fe­(CN)_6_]^3–/4–^ in 0.1 mol L^–1^ KCl was evaluated by varying the scan rate from 10 to 200 mV s^–1^ (Figure S8). Linear correlations were observed for *I*_pa_ vs the square root of the scan rate (ν^1/2^) (inset), characterizing a diffusion-controlled process, which is expected for the evaluated redox probe at the carbon electrode surface.

To evaluate the electrochemical performance of the proposed electrodes, CV and EIS measurements were carried out in the presence of 2.0 mmol L^–1^ [Fe­(CN)_6_]^3–/4–^ in 0.1 mol L^–1^ KCl, providing insight into electron transfer kinetics and overall electrochemical behavior and efficiency. The experiments were conducted using the proposed PDE (geometric area = 0.15 cm^2^), a commercial glassy carbon electrode (GCE; geometric area = 0.09 cm^2^), and a commercial carbon screen-printed electrode (C-SPE; geometric area = 0.11 cm^2^) for comparison. For a more meaningful comparison of the electrochemical responses, the peak currents were normalized by the geometric area of each electrode and expressed as current density (*j*).

For the PDE and GCE measurements, the same pseudoreference electrode (carbon pencil-drawing) was employed within the 3D-printed electrochemical cell described in this work. In the case of the GCE, the same cell configuration was maintained, with the PDE serving as the platform for the counter and reference electrodes, while the GCE was used as the working electrode and positioned as closely as possible to the original PDE working electrode location. In contrast, the C-SPE could not be accommodated within this configuration and was therefore evaluated using its integrated Ag pseudoreference and carbon counter electrodes. As expected, differences in reference electrode materials result in shifts in peak potentials.

To ensure a meaningful comparison, the peak-to-peak separation (Δ*E*_p_) was used as the primary metric, as it is independent of absolute potential values and thus appropriate for systems employing different reference electrodes, ensuring a valid comparison. For impedance measurements, experiments were performed at the half-wave potential (*E*_1_/_2_) of each system, minimizing the impact of potential shifts and enabling a consistent and reliable comparison of charge transfer kinetics among the evaluated electrodes. Representative data for the three electrode types are presented in Figure S9, and the main results of this study are summarized in Table 1.

**Table 1:** Comparison between the PDE, GCE, and C-SPE from Data Obtained by CV and EIS

Electrode	Geometric area (cm^2^)	*j*_anodic_ (μA cm^–2^)^a^	*j*_cathodic_ (μA cm^–2^)^a^	Δ*E*_p_ (mV)^a^	*R*_ct_ (kΩ)
PDE	0.15	309.41	322.26	119.00	0.45 ± 0.01
GCE	0.09	134.67	149.93	184.07	5.44 ± 0.25
C-SPE	0.11	230.74	213.22	172.98	2.51 ± 0.25

a Data obtained from cyclic voltammograms (*n* = 3) of the redox probes [Fe­(CN)­6]^3–/4–^in 0.1 mol L^–1^ KCl at 50 mV s^–1^ scan rate.

Cyclic voltammograms recorded in the presence of 2.0 mmol L^–1^ [Fe­(CN)_6_]^3–/4–^ in 0.1 mol L^–1^ KCl are presented in Figure S9A. The PDE exhibits markedly higher peak current densities compared to the GCE and C-SPE, reflecting an enhanced electrochemical response. Furthermore, the sharper and more well-defined peaks, together with the lower Δ*E*_p_ observed for the PDE, are indicative of improved reversibility of the redox process and more efficient charge transfer. In contrast, the broader peaks, lower current densities, and larger peak-to-peak separations observed for the GCE and C-SPE suggest comparatively slower electron transfer kinetics and reduced reversibility.

Nyquist plots obtained from EIS measurements in the presence of 2.0 mmol L^–1^ [Fe­(CN)_6_]^3–^^/^^4–^ in 0.1 mol L^–1^ KCl, recorded at the *E*_1/__2_ of each system, are presented in Figure S9B. The PDE exhibits the smallest high-frequency semicircle and the lowest *R*_ct_ among the evaluated electrodes. In contrast, the C-SPE shows intermediate *R*_ct_ values, while the GCE presents the highest *R*_ct_, indicating comparatively less favorable charge transfer kinetics. The equivalent circuit models used to fit the EIS data and extract the *R*_ct_ values for each system are presented in Figure S10.

These results are consistent with the CV data, confirming the superior electrochemical performance of the PDE. Although all electrodes exhibit features associated with diffusion-controlled processes, the significantly lower *R*_ct_ observed for the PDE highlights its enhanced charge transfer efficiency and suitability for electroanalytical applications, particularly in the detection of biological analytes.

Overall, the CV and EIS results demonstrate that the PDE presents improved electrochemical performance, characterized by enhanced redox reversibility and reduced charge-transfer resistance, supporting its potential as a miniaturized and disposable electrochemical sensor.

The electrochemical properties of the PDE were further explored through CV measurements and capacitance analysis. CV measurements were conducted from 0.0 to +0.3 V (vs pseudocarbon) at scan rates between 5 and 30 mV s^–1^ in 0.1 mol L^–1^ KCl (Figure S11A). The double-layer capacitance (*C*_dl_) was estimated by plotting the peak current density at +0.15 V (vs pseudocarbon and normalized by geometric area) as a function of scan rate (Figure S11B). The linear dependence of current density on scan rate indicates that the response is predominantly capacitive, reflecting a high electroactive surface area and reinforcing the electrode’s suitability for sensitive electroanalytical measurements.

### Detection of Biologically Relevant Analytes

The performance of the proposed electrode was investigated with biologically relevant molecules. CV experiments were conducted for dopamine, nitrite, paracetamol, and uric acid, and the representative responses are shown in Figure 4A–D. Different supporting electrolytes and pH conditions were employed for each analyte, based on previously reported optimal conditions for their electrochemical detection.^53–56^

**Figure 4:**
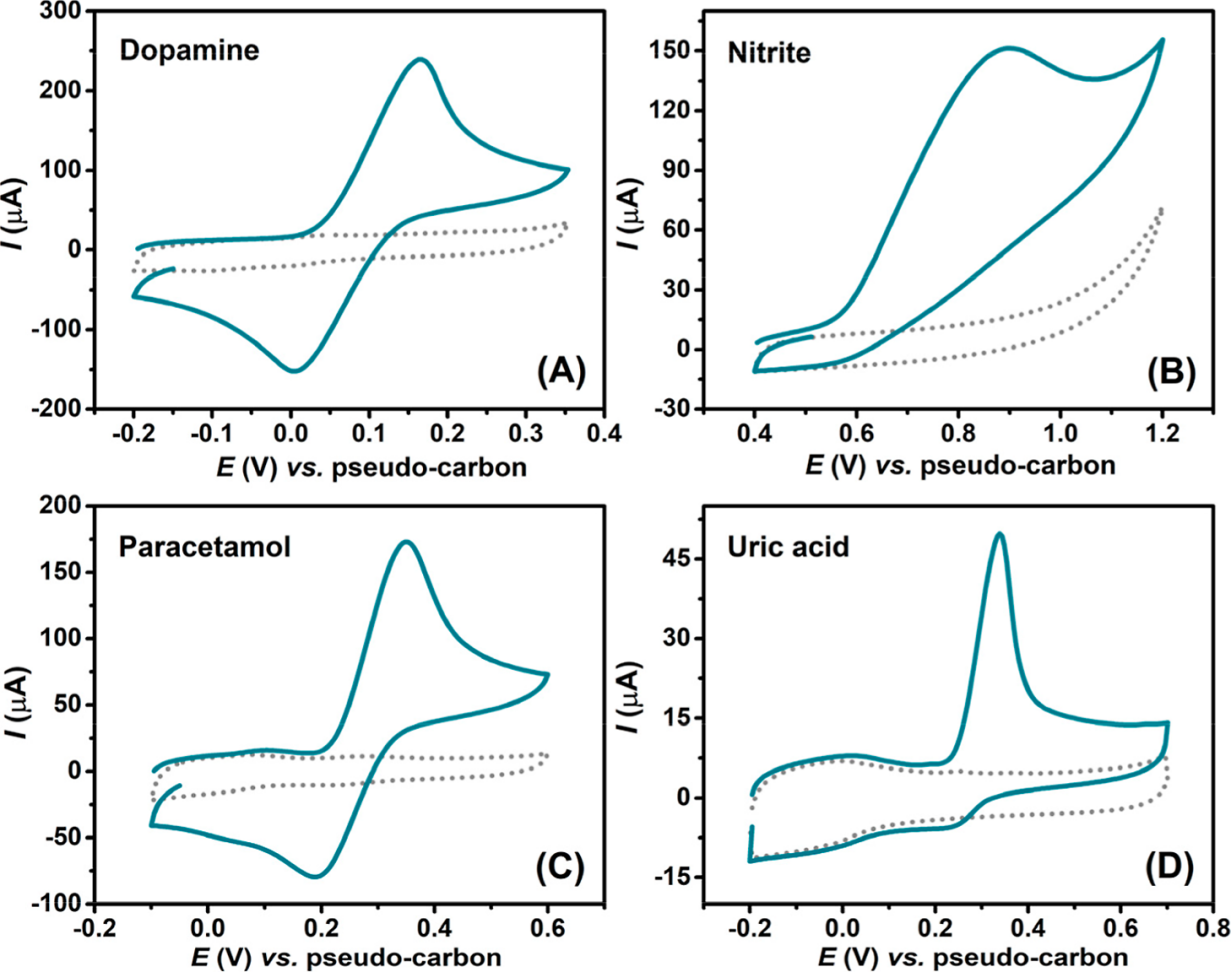
Cyclic voltammograms recorded in the presence of: (A) 2 mmol L^–1^ dopamine in 0.1 mol L^–1^ HClO_4_, (B) 1 mmol L^–1^ nitrite in 0.1 mol L^–1^ phosphate buffer (pH = 7.0), (C) 2 mmol L^–1^ paracetamol in acetate buffer (pH = 4.0), (D) 2 mmol L^–1^ uric acid in BR buffer (pH = 7.0). CV conditions: scan rate = 50 mV s^–1^; step potential = 5 mV. The gray/dotted line represents the baseline measurement with the matrix only, without the analyte.

The voltammograms shown in Figure 4 demonstrate that the proposed electrode provides well-defined electrochemical responses for all investigated analytes. Dopamine, paracetamol and uric acid exhibit clear oxidation peaks with good peak definition and low background current, indicating efficient electron-transfer kinetics at the graphite surface. As expected, nitrite displays only an anodic peak due to its irreversible oxidation mechanism. The magnitude and shape of the signals suggest that the electrode maintains stable performance across distinct buffer systems and pH values, confirming its applicability to molecules with diverse redox behaviors. These results highlight the versatility of the fabricated electrode for sensing biologically relevant compounds and reinforce its potential for use in analytical applications.

### Detection of Uric Acid

As proof of concept, the determination of uric acid was performed using the proposed electrochemical device. First, the influence of the solution pH on the electrochemical response of UA was investigated to evaluate the applicability of the proposed sensor. Figure S9 presents the cyclic voltammograms recorded at different pH values (Figure S12A), together with the plots of peak current (*I*_p_) and peak potential (*E*_p_) as a function of pH (Figure S12B). A clear shift of the anodic peak potential toward less positive values was observed with increasing pH, accompanied by a gradual decrease in peak current. The linear dependence of *E*_p_ on pH, with a slope close to −59 mV pH^–1^. Additionally, the higher peak currents obtained in acidic medium suggest more favorable electron transfer kinetics under these conditions. These findings confirm that the electrochemical oxidation of uric acid (at a concentration of 1.0 mmol L^–1^, supporting electrolyte: BR buffer, scan rate: 50 mV s^–1^) occurs through a proton-coupled electron transfer mechanism, strongly influenced by the solution pH.

The effect of scan rate on the voltammetric response of uric acid was investigated (Figure S13). As shown in Figure S13A, the peak current increased with increasing scan rate. A strong linear relationship between peak current (*I*_p_) and scan rate (ν) was observed (*r* = 0.999, Figure S13B), whereas a less satisfactory linearity was obtained for the *I*_p_ versus ν^1^´^/2^ relationship (*r* = 0.986, Figure S13C). This behavior indicates that the oxidation process is predominantly adsorption-controlled. Furthermore, the log–log plot of *I*_p_ versus ν (Figure S13D) yielded a slope of 1.2, which deviates from the theoretical value of 0.5 expected for diffusion-controlled processes and approaches the value of 1.0 typically associated with surface-controlled mechanisms. This result provides additional evidence that uric acid oxidation is primarily governed by a diffusionless, adsorption-controlled process.^57,58^ The slope value (∼1.2) exceeds the ideal theoretical value expected for a purely adsorption-controlled process, likely reflecting nonideal contributions such as capacitive currents, surface heterogeneity, or minor ohmic effects at higher scan rates.

Different electrochemical techniques were evaluated for this purpose, including differential pulse voltammetry (DPV) and square wave voltammetry (SWV) (data not shown). DPV proved to be the most suitable technique for UA determination, providing a well-defined peak current and lower background current compared to SWV. Therefore, it was selected as the analytical technique. Subsequently, DPV was subjected to optimization studies. Modulation time (Figure S14A,B), potential increment (Figure S14C,D), and amplitude (Figure S14E,F) were evaluated to obtain the best UA electrochemical response. Table S1 presents the ranges tested for each parameter and the conditions selected as optimal.

Next, a calibration curve was constructed under the optimized parameters for UA over a concentration range of 5.0 to 40.0 μmol L^–1^ (Figure S15). Figure S15B shows the corresponding calibration plot. The linear regression equation is *y* = 0.3588*x* + 0.3409 with a correlation coefficient (*r*) of 0.999. The limits of detection (LOD) and quantification (LOQ) were 0.2 and 0.6 μmol L^–1^, respectively. LOD and LOQ values were calculated, according to IUPAC guidelines (LOD = 3.3σ/s and LOQ = 10σ/s) where σ is standard deviation of the intercept, and *s* is the sensitivity obtained through the slope of the analytical curve.

The inter- and intraelectrode precision of the proposed method were also evaluated. Ten sequential electrochemical measurements were performed to assess repeatability (Figure S16), revealing an RSD of only 3.2% for *I*_pa_, indicating a good precision of the proposed method. To evaluate reproducibility, the study was conducted with three independent electrodes, resulting in an RSD of 4.7% for *I*_pa_. These findings confirm that the proposed sensors exhibit acceptable repeatability and reproducibility for the molecule of interest.

Table 2 summarizes several studies reporting UA determination using different carbon electrodes, considering their respective analytical techniques, detection limits (LOD), and linear ranges. The proposed sensor exhibited a linear range and LOD comparable to other disposable sensors reported in the literature. It is important to highlight that no electrode surface modification was required, and the device is miniaturized, requiring only a small volume of solution for the analyses.

**Table 2:** Comparison between This Work and Other Works Related to the Electrochemical Analysis of UA^a^

electrode	technique	LOD (mol L^–1^)	linear range (mol L^–1^)	reference
Pd–Pt/OMC/SPCE	amperometry	0.3	0.25–800	^63^
AuNPs/SPE	amperometry	15	20–200	^64^
PtNPs/MWCNT/SPCE	SWV	0.5	5–690	^65^
DMF/SPCE	DPV	0.2	5–500	^66^
SPCE	DPV	1.9	10–80	^67^
Co_3_O_4_–ERGO/SPE	DPV	1.5	5–500	^68^
SPCE-PEDOT:PSS	DPV	1.6	20–100	^69^
GS	DPV	0.08	1.0–400.0	^56^
PDE	DPV	0.2	5.0–40.0	This work

a Pd–Pt/OMC/SPCE: screen-printed carbon electrode modified with ordered mesoporous carbon decorated with a three-dimensional electrodeposited dendrite nanostructure of palladium–platinum; AuNPs/SPE: Gold nanoparticle nanocomposite modified screen-printed electrode; PtNPs/MWCNT/SPCE: screen-printed carbon electrodes modified with multiwalled carbon nanotubes and platinum nanoparticles; DMF/SPCE: DMF modified screen printed carbon electrodes; SPCE: screen-printed carbon electrode; Co_3_O_4_–ERGO/SPE: Co_3_O_4_ nanoparticles and electrochemically reduced graphene oxide on a screen-printed electrode; SPCE-PEDOT:PSS: screen-printed carbon electrode modified with poly­(3,4-ethylenedioxythiophene):poly­(styrenensulfonate); GS: graphite sheet.

Although carbon-based electrodes offer advantages such as simplicity, low cost, and ease of fabrication, their inherent lack of selectivity and limited catalytic activity can restrict performance in more complex analytical environments. In contrast, alternative sensing materials, such as metal–organic frameworks (MOFs), zeolites, and other structured composites, have been explored to improve selectivity and sensitivity in electrochemical sensing; however, these approaches often involve more complex fabrication procedures and higher cost.^59–62^

In contrast, advanced approaches based on modified carbon materials, such as plasma-treated graphite sheets electrodes,^56^ have demonstrated improved analytical performance for uric acid detection; however, these strategies require additional processing steps and specialized equipment, increasing the complexity of fabrication. In this context, the proposed platform offers a balanced compromise between analytical performance, simplicity, and sustainability, making it particularly suitable for disposable and point-of-care applications.

The proposed sensors were applied for the quantification of UA in synthetic saliva and synthetic urine samples (Figure 5A,B), and the results obtained are presented in Table 3. Good recovery values were also obtained for the spiked synthetic samples. The recovery ranges from 92 to 108%, indicating the sensor’s applicability for accurately determining uric acid in synthetic samples.

**Figure 5:**
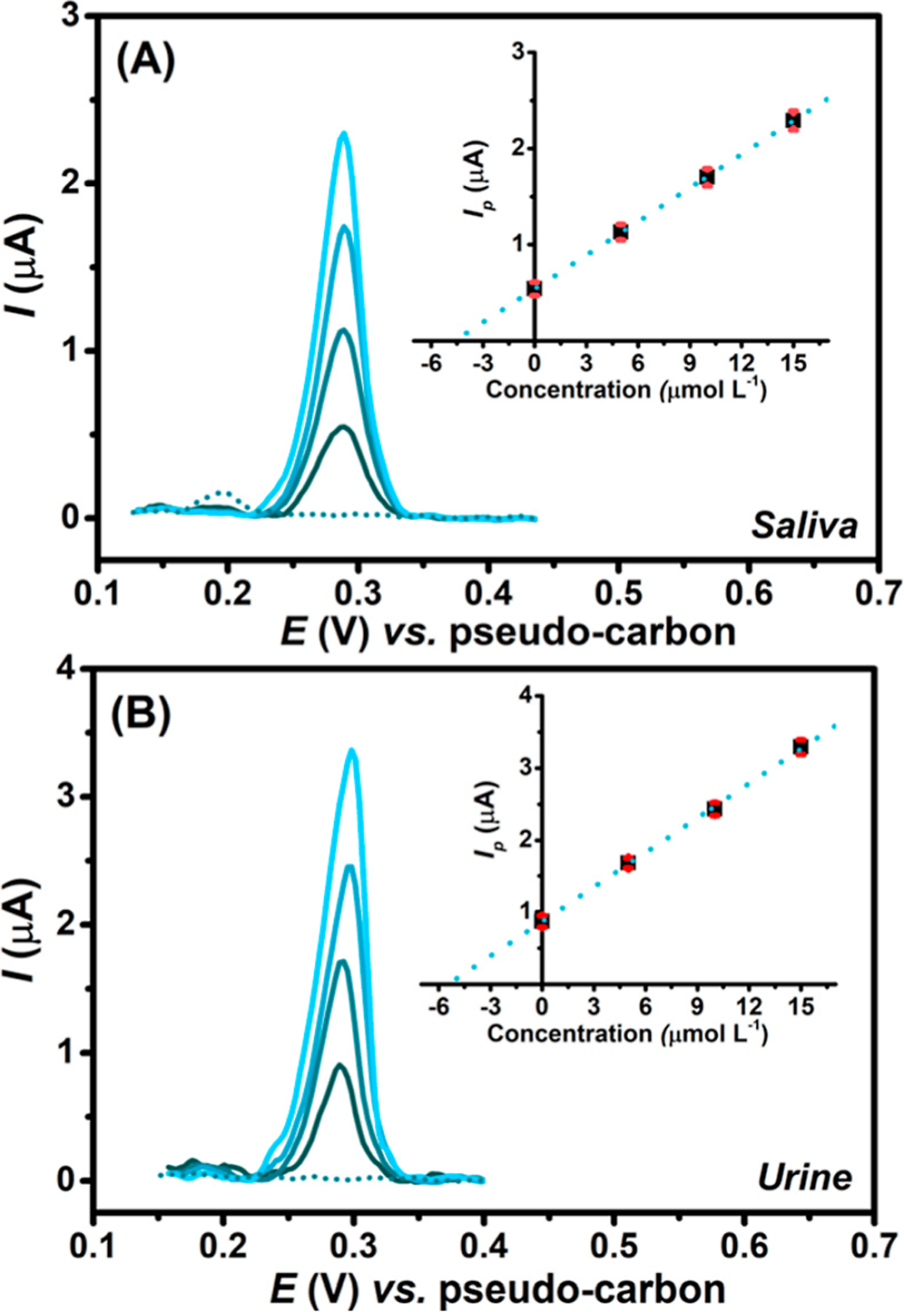
DPV voltammograms and respective standard addition curve (results for *n* = 3 measurements) obtained during one of the recoveries of UA performed in synthetic samples: (A) saliva and (B) urine. DPV conditions: modulation time = 80 ms; pulse amplitude = 50 mV; step potential = 3 mV. Supporting electrolyte: BR buffer (pH = 2.0).

**Table 3:** Recoveries of UA from Spiked Tablets Using the Proposed Electrochemical Method

sample	added (mol L^–1^)	found (mol L^–1^)	recovery (%)
saliva	5.0	4.6 ± 0.2	92 ± 4
urine	5.0	5.4 ± 0.3	108 ± 6

Finally, a quantitative selectivity study was carried out to evaluate the potential interference from common coexisting species, including ascorbic acid, glucose, urea, and dopamine under mixture conditions. The results demonstrated that the presence of these interfering compounds caused signal variations lower than 10%, indicating minimal impact on the analytical response. Additionally, recovery errors were also below 10%, confirming the high selectivity of the proposed sensor. These findings are presented in Figure S17.

## Conclusions

In this work, a simple, low-cost, and sustainable electrochemical platform based on pencil-drawn electrodes fabricated on an AM substrate was successfully developed for the determination of uric acid. The proposed device requires no surface modification, chemical treatment, or specialized instrumentation, relying solely on graphite deposition using a conventional pencil. Structural and electrochemical characterization confirmed that the AM substrate provides adequate roughness and stability for efficient graphite transfer, enabling the fabrication of reproducible and mechanically robust electrodes.

The sensor exhibited excellent electrochemical performance, with high reproducibility, low charge transfer resistance, and a capacitive profile consistent with a large electroactive area. Differential pulse voltammetry enabled sensitive UA quantification, offering a linear range of 5.0–40.0 μmol L^–1^ and a detection limit of 0.2 μmol L^–1^, comparable to or better than several modified disposable electrodes reported in the literature. Importantly, the electrode maintained stable performance without surface fouling, demonstrating consistent repeatability and reproducibility.

The PDE was successfully applied to the analysis of uric acid in synthetic saliva and urine, providing satisfactory recovery values (92–108%), which confirms its reliability for biological matrices. While more complex electrode modification strategies have been reported to enhance analytical performance, such approaches often require additional fabrication steps and specialized equipment. In contrast, the present platform prioritizes simplicity, accessibility, and sustainability, which are key factors for practical implementation in decentralized and resource-limited settings. These results highlight the potential of pencil-drawn electrodes as low-cost, eco-friendly, and accessible sensing tools, particularly suited for point-of-care testing and resource-limited environments. Overall, the PDE on stereolithography AM substrate represents a promising platform for the development of sustainable analytical devices and may be extended to the detection of other clinically and environmentally relevant analytes.

## Supplementary Material



## Notes

AFM measurements of the electrode surface as well as cyclic voltammetry studies addressing electrode reproducibility (different pencil batches, analysts, and substrates) and repeatability, contact angle measurements, and the influence of reference electrodes. Scan rate studies and electrochemical behavior of the redox probe were evaluated through *I*_p_ vs. *v*, *I*_p_ vs *v* ^1^´^2^, and log *I*_p_ vs log *v* relationships. Comparative electrochemical performance of the pencil-drawn electrode (PDE), glassy carbon electrode (GCE), and screen-printed electrode (SPE) was assessed by cyclic voltammetry and electrochemical impedance spectroscopy (EIS), including Nyquist plots and double-layer capacitance estimation. Additional voltammetric investigations include the effect of pH on uric acid (UA) oxidation, scan rate studies for UA, and optimization of differential pulse voltammetry (DPV) parameters (modulation time, step potential, and amplitude). Calibration curves, repeatability, stability and selectivity results, and successive measurements are also presented (PDF)

## References

[1] Sajeevan A., Sukumaran R. A., Panicker L. R., Kotagiri Y. G. (2025). Trends in Ready-to-Use
Portable Electrochemical Sensing Devices for Healthcare Diagnosis. Microchim. Acta.

[2] Suhito I. R., Koo K.-M., Kim T.-H. (2021). Recent
Advances in Electrochemical
Sensors for the Detection of Biomolecules and Whole Cells. Biomedicines.

[3] Copur S., Demiray A., Kanbay M. (2022). Uric Acid
in Metabolic Syndrome:
Does Uric Acid Have a Definitive Role?. Eur.
J. Int. Med..

[4] Vernerová A., Krčmová L. K., Heneberk O., Radochová V., Strouhal O., Kašparovský A., Melichar B., Švec F. (2021). Chromatographic Method for the Determination
of Inflammatory
Biomarkers and Uric Acid in Human Saliva. Talanta.

[5] Wijemanne N., Soysa P., Wijesundara S., Perera H. (2018). Development and Validation
of a Simple High Performance Liquid Chromatography/UV Method for Simultaneous
Determination of Urinary Uric Acid, Hypoxanthine, and Creatinine in
Human Urine. Int. J. Anal. Chem..

[6] Wang J. (2002). Portable Electrochemical
Systems. TrAC, Trends Anal. Chem..

[7] He Q., Wang B., Liang J., Liu J., Liang B., Li G., Long Y., Zhang G., Liu H. (2023). Research on the Construction
of Portable Electrochemical Sensors for Environmental Compounds Quality
Monitoring. Mater. Today Adv..

[8] Naseri M., Ziora Z. M., Simon G. P., Batchelor W. (2022). ASSURED-compliant
Point-of-care Diagnostics for the Detection of Human Viral Infections. Rev. Med. Virol..

[9] A Global Health Strategy for 2025–2028 - Advancing Equity and Resilience in a Turbulent World; World Health Organization, 2025.

[10] Wang L., Pumera M. (2021). Recent Advances of 3D Printing in
Analytical Chemistry:
Focus on Microfluidic, Separation, and Extraction Devices. TrAC, Trends Anal. Chem..

[11] Ramos D. L. O., de Faria L. V., Alves D. A. C., Muñoz R. A. A., dos Santos W. T. P., Richter E. M. (2023). Electrochemical
Platform Produced by 3D Printing for Analysis of Small Volumes Using
Different Electrode Materials. Talanta.

[12] Katseli V., Economou A., Kokkinos C. (2019). Single-Step Fabrication
of an Integrated
3D-Printed Device for Electrochemical Sensing Applications. Electrochem. Commun..

[13] Selemani M. A., Cenhrang K., Azibere S., Singhateh M., Martin R. S. (2024). 3D Printed Microfluidic Devices with
Electrodes for
Electrochemical Analysis. Anal. Methods.

[14] von
Zuben T. W., Camargo J. R., Rocha R. G., dos Santos P. L., Richter E. M., Janegitz B. C., Munoz R. A. A., Bonacin J. A. (2025). Advances
and the Growing Role of Additive Manufacturing in the Development
of 3D-Printed Electrochemical Sensors. Electrochim.
Acta.

[15] Orzari L. O., Kalinke C., Silva-Neto H. A., Rocha D. S., Camargo J. R., Coltro W. K. T., Janegitz B. C. (2025). Screen-Printing
vs Additive Manufacturing
Approaches: Recent Aspects and Trends Involving the Fabrication of
Electrochemical Sensors. Anal. Chem..

[16] Bernalte E., Foster C., Brownson D., Mosna M., Smith G., Banks C. (2016). Pencil It in: Exploring
the Feasibility of Hand-Drawn Pencil Electrochemical
Sensors and Their Direct Comparison to Screen-Printed Electrodes. Biosensors.

[17] Ataide V. N., Ameku W. A., Bacil R. P., Angnes L., de Araujo W. R., Paixão T. R. L. C. (2021). Enhanced
Performance of Pencil-Drawn
Paper-Based Electrodes by Laser-Scribing Treatment. RSC Adv..

[18] Ferreira B., Arantes I. V. S., Saraiva D. P. M., Pradela-Filho L. A., Bertotti M., Paixão T. R. L.
C. (2024). Commercial Ink-Coated
PVC: No Longer Abrading Conventional PVC Surfaces for Electrode Fabrication
Using Pencil Drawing. Microchem. J..

[19] Dossi N., Petrazzi S., Terzi F., Toniolo R., Bontempelli G. (2019). Electroanalytical
Cells Pencil Drawn on PVC Supports and Their Use for the Detection
in Flexible Microfluidic Devices. Talanta.

[20] Foster C. W., Brownson D. A. C., Ruas
de Souza A. P., Bernalte E., Iniesta J., Bertotti M., Banks C. E. (2016). Pencil It in: Pencil Drawn Electrochemical
Sensing Platforms. Analyst.

[21] Orzari L. O., de Araujo Andreotti I. A., Bergamini M. F., Marcolino L. H., Janegitz B. C. (2018). Disposable Electrode Obtained by
Pencil Drawing on Corrugated Fiberboard Substrate. Sens. Actuators B Chem..

[22] de
Moraes N. C., Daakour R. J. B., Pedão E. R., Ferreira V. S., da Silva R. A. B., Petroni J. M., Lucca B. G. (2023). Electrochemical
Sensor Based on 3D-Printed Substrate by Masked Stereolithography (MSLA):
A New, Cheap, Robust and Sustainable Approach for Simple Production
of Analytical Platforms. Microchim. Acta.

[23] Ahmad K., Mobin S. M. (2019). Shape Controlled
Synthesis of High Surface Area MgO
Microstructures for Highly Efficient Congo Red Dye Removal and Peroxide
Sensor. J. Environ. Chem. Eng..

[24] Abdolmohammad-Zadeh H., Zamani-Kalajahi M. (2020). In Situ Generation of H2O2 by a Layered
Double Hydroxide
as a Visible Light Nano-Photocatalyst: Application to Bisphenol A
Quantification. Microchem. J..

[25] Cui Y., Lai B., Tang X. (2019). Microbial Fuel Cell-Based Biosensors. Biosensors.

[26] Romonti D. E., Gomez Sanchez A. V., Milošev I., Demetrescu I., Ceré S. (2016). Effect of
Anodization on the Surface Characteristics
and Electrochemical Behaviour of Zirconium in Artificial Saliva. Mater. Sci. Eng. C.

[27] Brooks T., Keevil C. W. (1997). A Simple Artificial
Urine for the Growth of Urinary
Pathogens. Lett. Appl. Microbiol..

[28] https://www.geogebra.org/classic (accessed 28th May 2026).

[29] Liu Y., Tang Z., Zhu J. (2022). Synergistic
Flame Retardant Effect
of Aluminum Hydroxide and Ammonium Polyphosphate on Epoxy Resin. J. Appl. Polym. Sci..

[30] Ferrari A. C., Basko D. M. (2013). Raman Spectroscopy as a Versatile Tool for Studying
the Properties of Graphene. Nat. Nanotechnol..

[31] Jorio A., Ferreira E. H. M., Moutinho M. V. O., Stavale F., Achete C. A., Capaz R. B. (2010). Measuring Disorder
in Graphene with the G and D Bands. physica
status solidi (b).

[32] Beams R., Gustavo Cançado L., Novotny L. (2015). Raman Characterization
of Defects and Dopants in Graphene. J. Phys.:
Condens. Matter.

[33] Kwiecinska B., Suárez-Ruiz I., Paluszkiewicz C., Rodriques S. (2010). Raman Spectroscopy
of Selected Carbonaceous Samples. Int. J. Coal
Geol..

[34] Papanai G. S., Sharma I., Gupta B. K. (2020). Probing
Number of Layers and Quality
Assessment of Mechanically Exfoliated Graphene via Raman Fingerprint. Mater. Today Commun..

[35] Eckmann A., Felten A., Mishchenko A., Britnell L., Krupke R., Novoselov K. S., Casiraghi C. (2012). Probing the Nature of Defects in
Graphene by Raman Spectroscopy. Nano Lett..

[36] Srinivas S., Senthil Kumar S. M., Senthil Kumar A. (2023). Edge and Basal Plane Anisotropy of
a Preanodized Pencil Graphite Electrode Surface Revealed Using Scanning
Electrochemical Microscopy and Electrocatalytic Dopamine Oxidation
as a Molecular Probe. Langmuir.

[37] Garboczi E. J., Snyder K. A., Douglas J. F., Thorpe M. F. (1995). Geometrical Percolation
Threshold of Overlapping Ellipsoids. Phys. Rev.
E:Stat., Nonlinear, Soft Matter Phys..

[38] Zou J., Yu Z., Pan Y., Fang X., Ou Y. (2002). Conductive Mechanism
of Polymer/Graphite Conducting Composites with Low Percolation Threshold. J. Polym. Sci. B Polym. Phys..

[39] Stankovich S., Dikin D. A., Dommett G. H. B., Kohlhaas K. M., Zimney E. J., Stach E. A., Piner R. D., Nguyen S. T., Ruoff R. S. (2006). Graphene-Based
Composite Materials. Nature.

[40] Stauffer, D. ; Aharony, A. Introduction To Percolation Theory; Taylor & Francis, 2018.

[41] Brigandi P.
J., Cogen J. M., Pearson R. A. (2014). Electrically Conductive Multiphase
Polymer Blend Carbon-based Composites. Polym.
Eng. Sci..

[42] Yu L., Zhang Y.-H., Shang J., Ke S.-M., Tong W., Shen B., Huang H.-T. (2012). Electrical
and Dielectric Properties
of Exfoliated Graphite/Polyimide Composite Films with Low Percolation
Threshold. J. Electron. Mater..

[43] Zhang S., Ukrainczyk N., Zaoui A., Koenders E. (2024). Electrical Conductivity
of Geopolymer-Graphite Composites: Percolation, Mesostructure and
Analytical Modeling. Constr. Build. Mater..

[44] Eckmann A., Felten A., Mishchenko A., Britnell L., Krupke R., Novoselov K. S., Casiraghi C. (2012). Probing the Nature of Defects in
Graphene by Raman Spectroscopy. Nano Lett..

[45] Velický M., Toth P. S., Woods C. R., Novoselov K. S., Dryfe R. A. W. (2019). Electrochemistry of the Basal Plane
versus Edge Plane
of Graphite Revisited. J. Phys. Chem. C.

[46] McCreery R. L. (2008). Advanced
Carbon Electrode Materials for Molecular Electrochemistry. Chem. Rev..

[47] Zhang G., Kirkman P. M., Patel A. N., Cuharuc A. S., McKelvey K., Unwin P. R. (2014). Molecular Functionalization of Graphite Surfaces: Basal
Plane versus Step Edge Electrochemical Activity. J. Am. Chem. Soc..

[48] Ganesan P., Vanaki Sh. M., Thoo K. K., Chin W. M. (2016). Air-Side Heat Transfer
Characteristics of Hydrophobic and Super-Hydrophobic Fin Surfaces
in Heat Exchangers: A Review. Int. Commun. Heat
Mass Transfer.

[49] Kozbial A., Li Z., Sun J., Gong X., Zhou F., Wang Y., Xu H., Liu H., Li L. (2014). Understanding the Intrinsic Water
Wettability of Graphite. Carbon N. Y..

[50] Jao W.-Y., Tseng L.-C., Lin Y., Chen Y.-C., Hu C.-C. (2024). Stability
Promotion of Carbon-Based Quasi-Reference Electrodes in Nonaqueous
Calcium Ion Electrolytes. J. Phys. Chem. C.

[51] Sophocleous M., Atkinson J. K. (2017). A Review of Screen-Printed
Silver/Silver Chloride (Ag/AgCl)
Reference Electrodes Potentially Suitable for Environmental Potentiometric
Sensors. Sens. Actuators A Phys..

[52] Søpstad S., Johannessen E. A., Seland F., Imenes K. (2018). Long-Term Stability
of Screen-Printed Pseudo-Reference Electrodes for Electrochemical
Biosensors. Electrochim. Acta.

[53] Cardoso R. M., Mendonça D. M. H., Silva W. P., Silva M. N. T., Nossol E., da Silva R. A. B., Richter E. M., Muñoz R. A. A. (2018). 3D Printing
for Electroanalysis: From Multiuse Electrochemical Cells to Sensors. Anal. Chim. Acta.

[54] Cardoso R. M., Silva P. R. L., Lima A. P., Rocha D. P., Oliveira T. C., do Prado T. M., Fava E. L., Fatibello-Filho O., Richter E. M., Muñoz R. A. A. (2020). 3D-Printed
Graphene/Polylactic Acid
Electrode for Bioanalysis: Biosensing of Glucose and Simultaneous
Determination of Uric Acid and Nitrite in Biological Fluids. Sens. Actuators B Chem..

[55] Shiroma L. Y., Santhiago M., Gobbi A. L., Kubota L. T. (2012). Separation and Electrochemical
Detection of Paracetamol and 4-Aminophenol in a Paper-Based Microfluidic
Device. Anal. Chim. Acta.

[56] Marra M. C., Di-Oliveira M., Rocha R. G., Terra T. R., Crapnell R. D., Banks C. E., Richter E. M., Muñoz R. A. A. (2026). Automated
Air Plasma-Assisted Functionalization of Graphite Electrodes for Enhanced
Electrochemical Sensing of Uric Acid. Microchim.
Acta.

[57] de
Oliveira F. M., dos Santos W. T. P. (2025). A Simple and Efficient Approach for
Simulation of Adsorbed Species in the Diffusionless Case: A New Look
on Chronoamperometry Data. Electrochim. Acta.

[58] Speiser, B. Numerical Simulations in Electrochemistry. In Encyclopedia of Applied Electrochemistry; Springer: New York: New York, NY, 2014; pp 1380–1385.

[59] Muthukumaran M. K., Govindaraj M., Kogularasu S., Sriram B., Raja B. K., Wang S.-F., Chang-Chien G.-P., Selvi J A. (2025). Recent Advances in
Metal-Organic Frameworks for Electrochemical Sensing Applications. Talanta Open.

[60] Damacet P., Shehayeb E. O., Monti S., Barcaro G., Mirica K. A. (2025). Redox-Active
Metal–Organic Framework Nanocrystals for the Simultaneous Adsorption,
Detection, and Detoxification of Heavy Metal Cations. ACS Appl. Mater. Interfaces.

[61] Walcarius A. (2026). Recent Advances
in Electrochemistry with Zeolitic Materials. Curr. Opin. Electrochem..

[62] Karagianni A., Tsierkezos N. G., Ntziouni A., Terrones M., Kordatos K. V. (2026). Carbon
Nanotubes as Electrochemical Sensors for Neurotransmitters: Synthesis,
Doping, and Applications. Carbon N. Y..

[63] Khumngern S., Choosang J., Thavarungkul P., Kanatharana P., Numnuam A. (2020). Flow Injection Enzyme-Free Amperometric
Uric Acid Sensor
Consisting of Ordered Mesoporous Carbon Decorated with 3D Pd-Pt Alloy
Nanodendrite Modified Screen-Printed Carbon Electrode. Microchem. J..

[64] Piedras J., Dominguez R. B., Gutiérrez J. M. (2021). Determination of Uric Acid in Artificial
Saliva with Compact AMP3291 Reader and Au Nanoparticles Modified Electrode. Chemosensors.

[65] Cardoso
Gomes-Junior P., Dias Nascimento E., Kenlderi de Lima Augusto K., Patelli Longatto G., Censi Faria R., Piccin E., Fatibello-Filho O. (2024). Voltammetric
Determination of Uric Acid Using a Miniaturized Platform Based on
Screen-Printed Electrodes Modified with Platinum Nanoparticles. Microchem. J..

[66] Metto M., Eramias S., Gelagay B., Washe A. P. (2019). Voltammetric Determination
of Uric Acid in Clinical Serum Samples Using DMF Modified Screen Printed
Carbon Electrodes. Int. J. Electrochem..

[67] Wahyuni W. T., Putra B. R., Heryanto R., Rohaeti E., Yanto D. H. Y., Fauzi A. (2021). A Simple Approach to Fabricate a
Screen-Printed Electrode
and Its Application for Uric Acid Detection. Int. J. Electrochem. Sci..

[68] Turkkan G., Bas S. Z., Atacan K., Ozmen M. (2021). An Electrochemical
Sensor Based on a Co _3_ O _4_ – ERGO Nanocomposite
Modified Screen-Printed Electrode for Detection of Uric Acid in Artificial
Saliva. Anal. Methods.

[69] Wahyuni W. T., Heryanto R., Rohaeti E., Fauzi A., Putra B. R. (2021). Uric Acid
Sensor Based on PEDOT:PSS Modified Screen-Printed Carbon Electrode
Fabricated with a Simple Painting Technique. J. Kim. Sains Apl..

