# Supplementary material for: Pencil-Drawn Electrode within Additively Manufactured Devices for Uric Acid Detection
Source: ACS Omega. 2026 Jun 1;11(23):34392–403. doi: 10.1021/acsomega.6c02346 (PMC13280844; doi:10.1021/acsomega.6c02346)
Supplement: Supplementary file 1 [file ao6c02346_si_001.pdf]

Pencil-drawn electrode within additively manufactured devices for uric acid detection

Mariana C. Marra[a], Marina Di-Oliveira[a], Amanda B. Nascimento[a], Raquel G. Rocha[a,b], Natália C. de Moraes[c], Eduardo M. Richter[a], Bruno G. Lucca[c], Rodrigo A. A. Muñoz[a*]

[a]*Federal University of Uberlândia, Chemistry Institute, 38400-902, Uberlândia, MG, Brazil*

[b]*University of Warwick, Department of Chemistry, CV48UW, Coventry, United Kingdom*

[c]*Federal University of Mato Grosso do Sul, Chemistry Institute, 79074-460, Campo Grande, MS, Brazil*

***Corresponding author:**

munoz@ufu.br

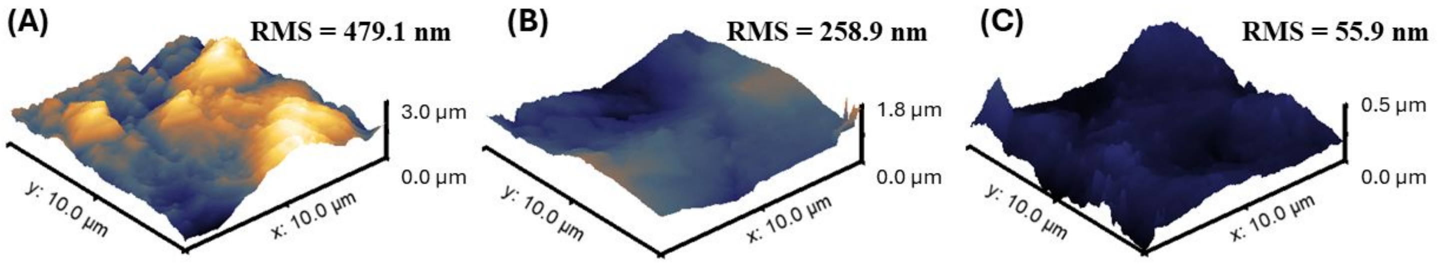

Figure S1: AFM topographic images of **(A)** bare stereolithography additively manufactured substrate, **(B)** pencil-drawn electrode (PDE) with a single graphite layer, and **(C)** PDE with 30 layers of redeposited graphite composite film. The corresponding root mean square (RMS) roughness values are indicated for each surface and were obtained using the same Z-scale, enabling reliable quantitative comparison. The color range in the 3D representations was normalized to the highest roughness condition to allow consistent visual comparison among the samples, while the Z-axis scales were kept as originally acquired for each surface to preserve the visualization of topographical features.

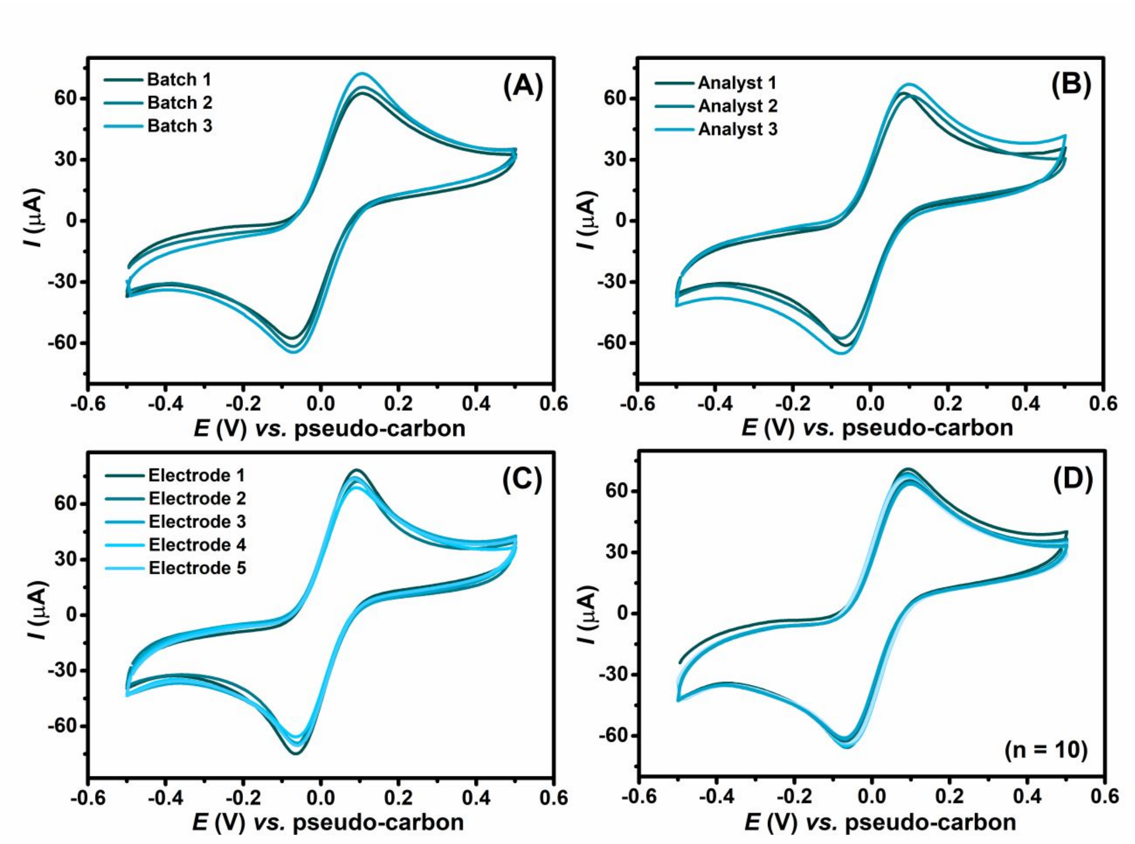

Figure S2: Cyclic voltammetry measurements performed using electrodes prepared with **(A)** three different batches of pencils, **(B)** three different analysts, **(C)** five different substrates, and **(D)** a repeatability study based on fifty consecutive scans. All measurements were carried out in the presence of 2.0 mmol L^{-1} $[Fe(CN)_6]^{3-/4-}$ in 0.1 mol L^{-1} KCl at a scan rate of 50 mV s^{-1}.

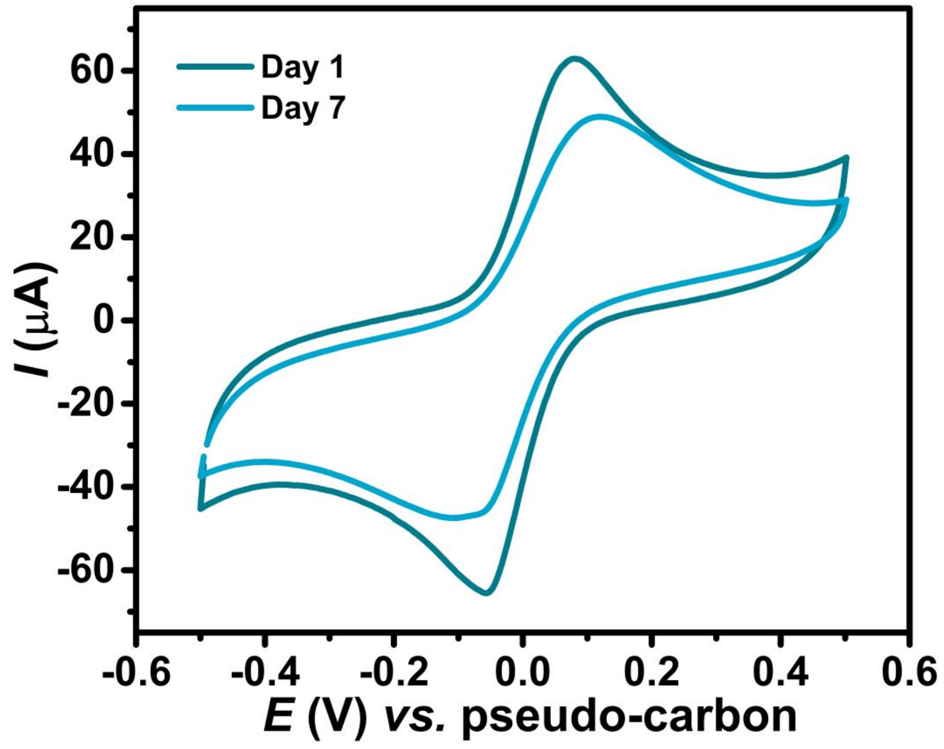

Figure S3: Cyclic voltammograms recorded for a single electrode on day 1 and day 7. Measurements were carried out in the presence of 2.0 mmol L^{-1} $[Fe(CN)_6]^{3-/4-}$ in 0.1 mol L^{-1} KCl at a scan rate of 50 mV s^{-1}.

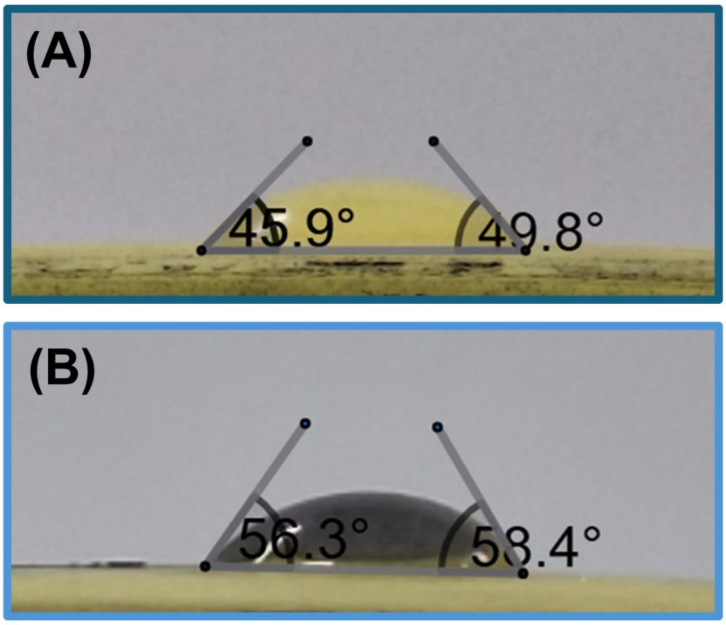

Figure S4: Contact angle measurements of **(A)** the substrate and **(B)** the PDE.

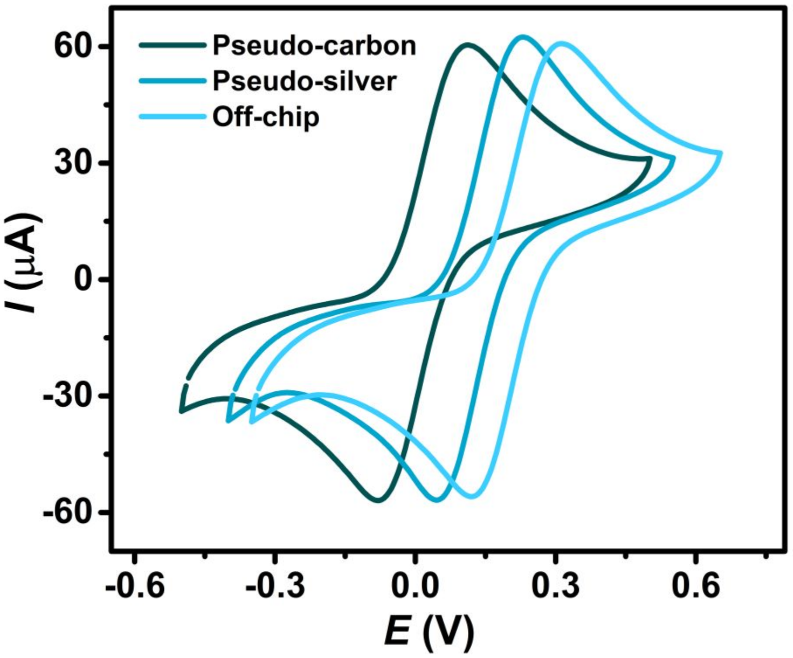

Figure S5: Cyclic voltammograms recorded using a pencil-drawn electrode (PDE) in the presence of 2.0 mmol L^{-1} $[Fe(CN)_6]^{3-/4-}$ in 0.1 mol L^{-1} KCl, employing different reference electrodes. **CV conditions**: scan rate = 50 mV s^{-1}; step potential = 5 mV.

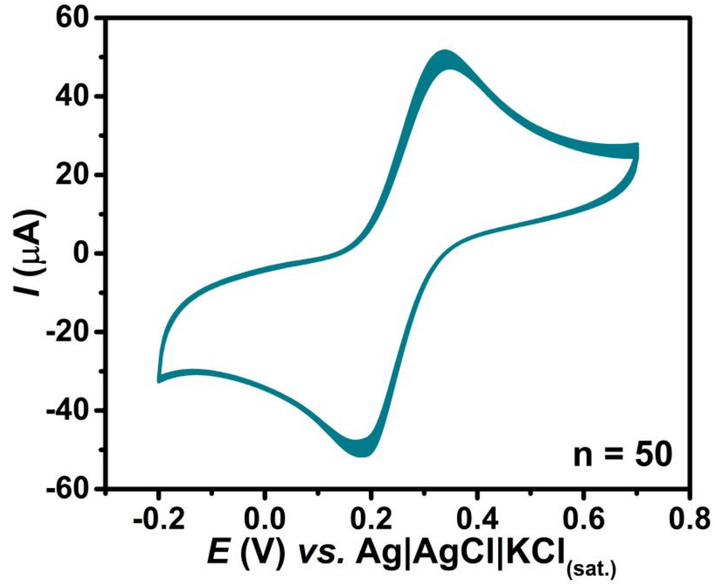

Figure S6: Cyclic voltammetry measurements performed for a repeatability study based on fifty consecutive scans using an external reference electrode. All measurements were carried out in the presence of 2.0 mmol L^{-1} $[Fe(CN)_6]^{3-/4-}$ in 0.1 mol L^{-1} KCl at a scan rate of 50 mV s^{-1}.

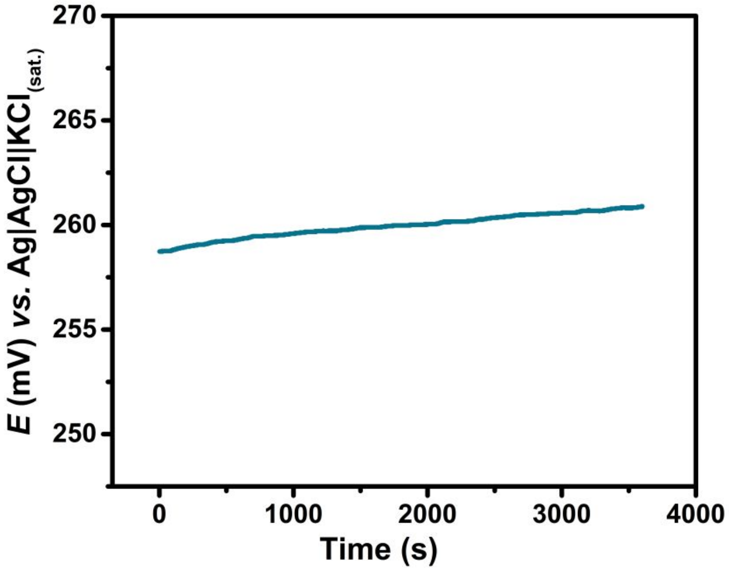

Figure S7: OCP measurements recorded for pseudo-carbon as the working electrode and $Ag/AgCl/KCl_{(sat.)}$ as reference electrode. All measurements were carried out in the presence of 2.0 mmol L^{-1} $[Fe(CN)_6]^{3-/4-}$ in 0.1 mol L^{-1} KCl at a scan rate of 50 mV s^{-1}.

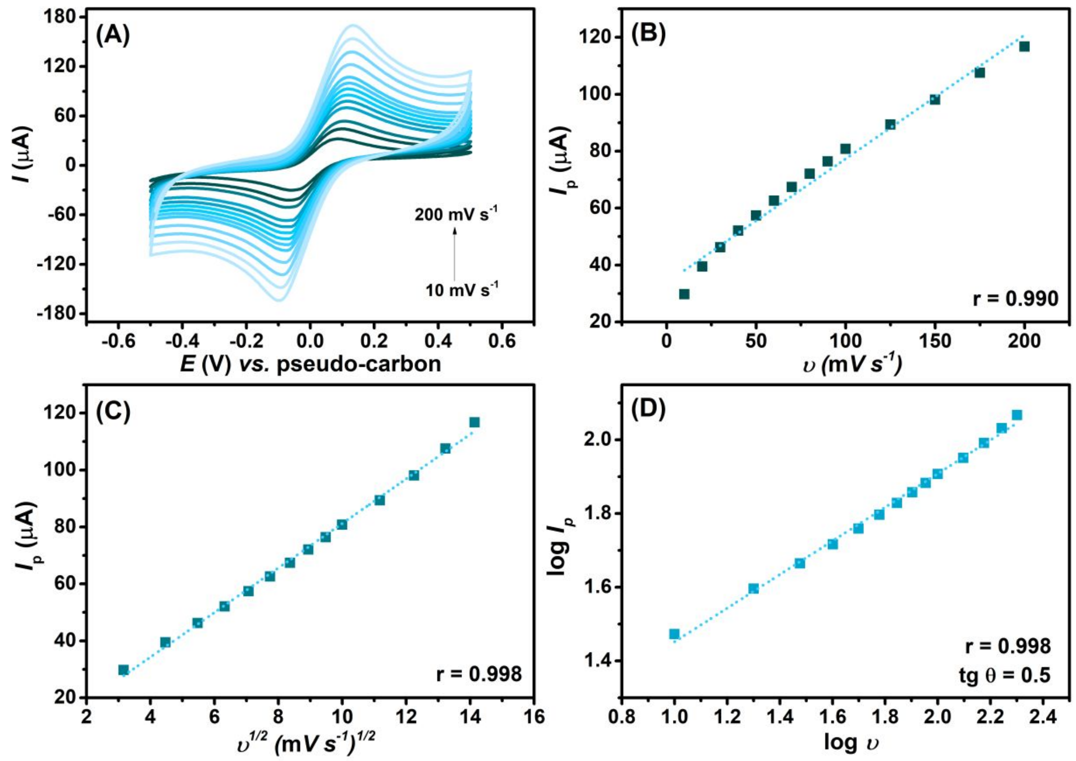

Figure S8: (A) Cyclic voltammograms recorded at various scan rates (10 to 200 mV s^{-1}) using the PDE in the presence of 2.0 mmol L^{-1} $[Fe(CN)_6]^{3-/4-}$ in 0.1 mol L^{-1} KCl. Plot of peak currents (I_p) *vs* **(B)** scan rate, or **(C)** square root of scan rate ($v^{1/2}$) **(D)** log I_p vs log scan rate.

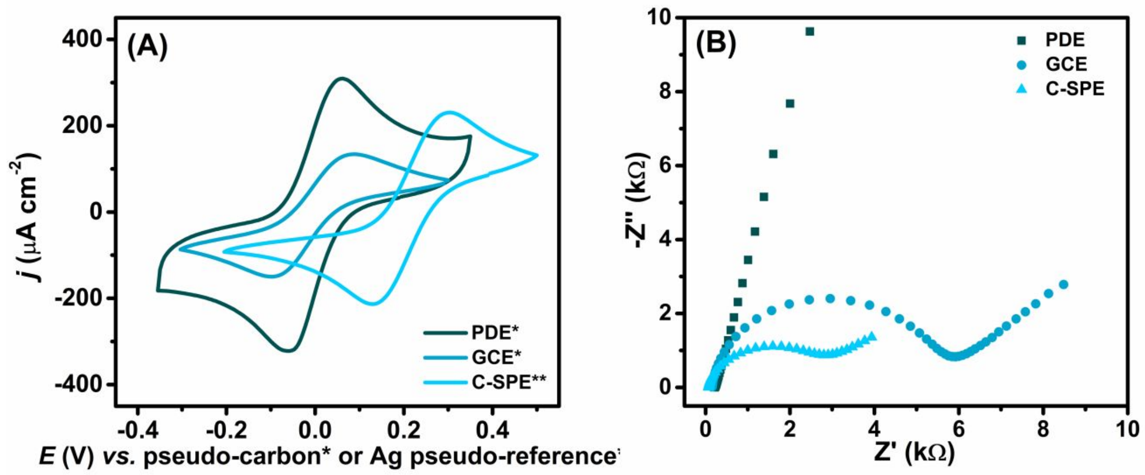

Figure S9: (A) Cyclic voltammograms recorded in the presence (solid lines) of 2.0 mmol L^{-1} $[Fe(CN)_6]^{3-/4-}$ in 0.1 mol L^{-1} KCl using PDE (Geometric area: 0.15 cm^2; dark blue lines), GCE (Geometric area: 0.09 cm^2; light green lines), and C-SPE (Geometric area:

0.11 cm²; light blue lines) as working electrodes. **(B)** Nyquist plots obtained from electrochemical impedance spectroscopy (EIS) in the same conditions of **(A)**. **CV conditions**: scan rate = 50 mV s^{-1}; step potential = 5 mV. The supporting electrolyte was 0.1 mol L^{-1} KCl.

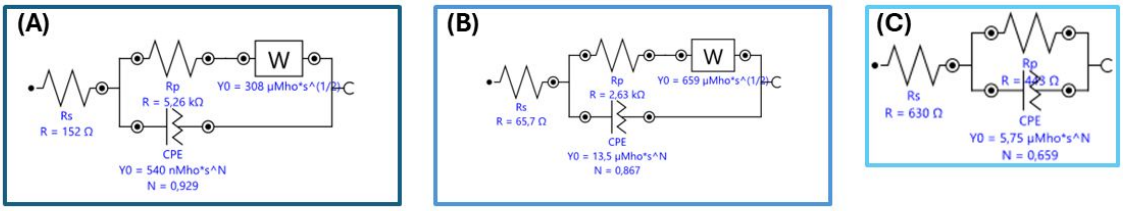

Figure S10: Randles equivalent circuit models employed for fitting the EIS data associated with the Nyquist spectra shown in Figure S5 for (A) GCE (geometric area: 0.09 cm²; light green), (B) C-SPE (geometric area: 0.11 cm²; light blue), and (C) PDE (geometric area: 0.15 cm²; dark blue).

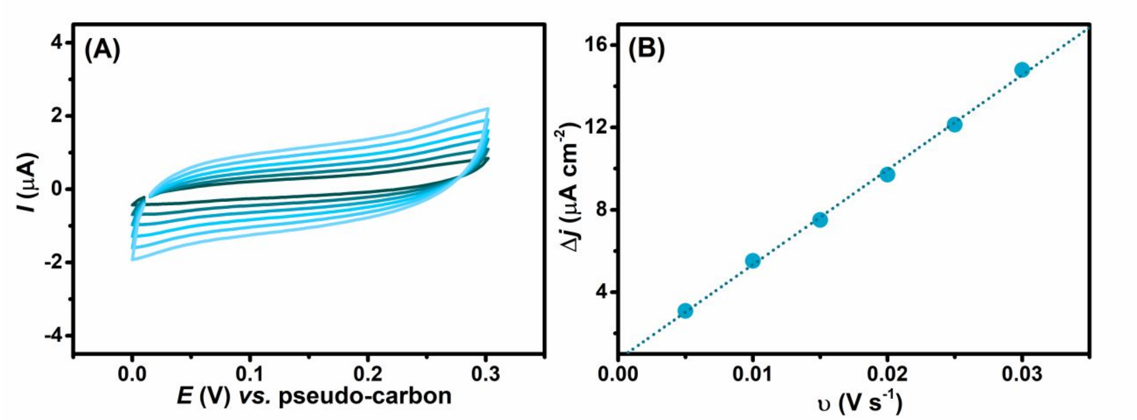

Figure S11: (A) Cyclic voltammograms recorded from 0.0 to +0.3 V *vs.* pseudo-carbon at scan rates from 5 to 30 mV s^{-1} using the PDE in 0.1 mol L^{-1} KCl solution. **(B)** Capacitance analysis: plots of Δj (peak current density measured at +0.15 V *vs.* pseudo-carbon, normalized by geometric area) as a function of scan rate, used to estimate the double-layer capacitance (C*dl*) of the PDE. **CV condition**: step potential = 5 mV.

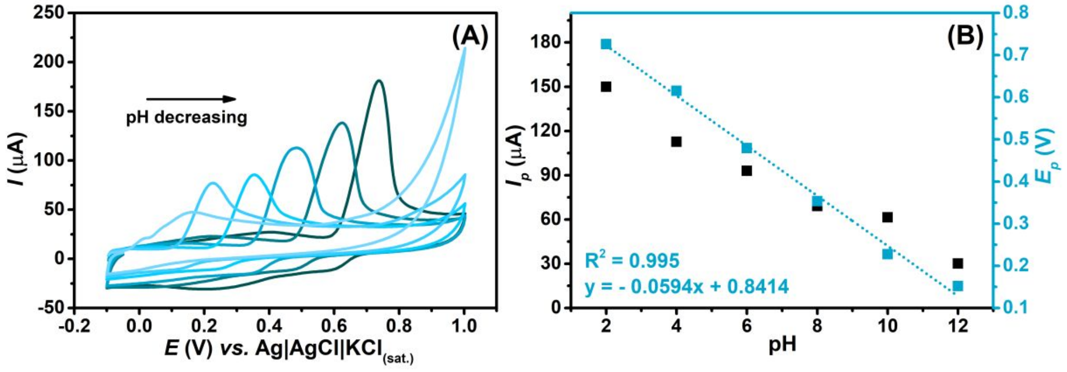

Figure S12: (A) Cyclic voltammograms of 1 mmol L⁻¹ UA recorded at different pH values (2.0 to 12.0). **(B)** Dependence of the peak currents and peak potentials on the pH. **CV conditions**: scan rate = 50 mV s^{-1}; step potential = 5 mV. Supporting electrolyte: BR buffer.

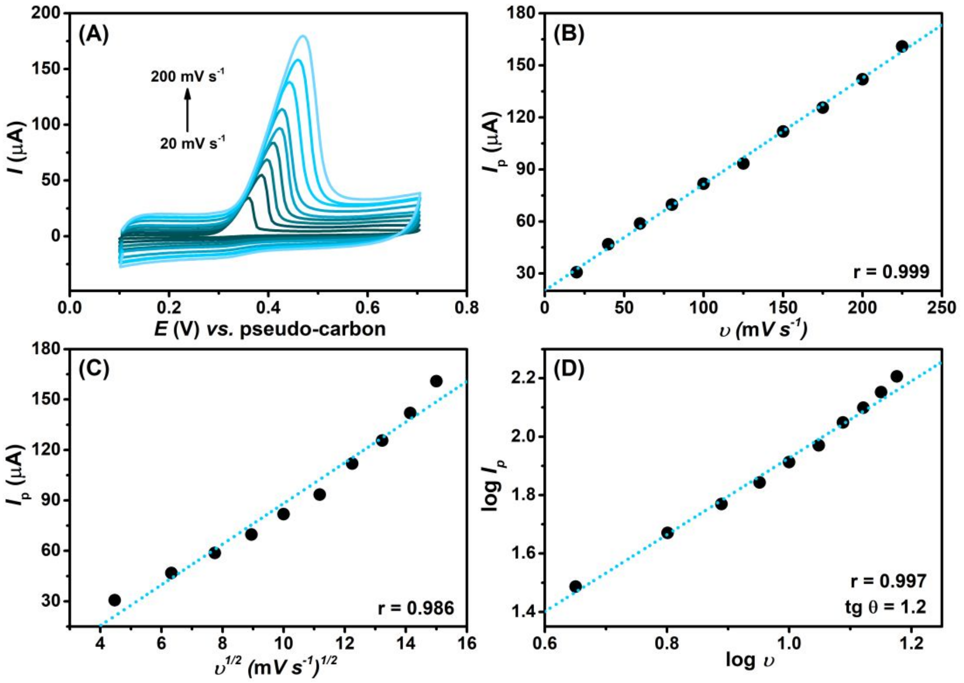

Figure S13: (A) Cyclic voltammograms recorded at 20 to 200 mV s^{-1} at the PDE. CVs were measured in the presence of 0.5 mmol L^{-1} UA in BR buffer (pH 2.0). Plot of peak

currents (I_p) *vs* **(B)** scan rate, or **(C)** square root of scan rate ($v^{1/2}$) **(D)** log I_p vs log scan rate. **CV condition**: step potential = 5 mV.

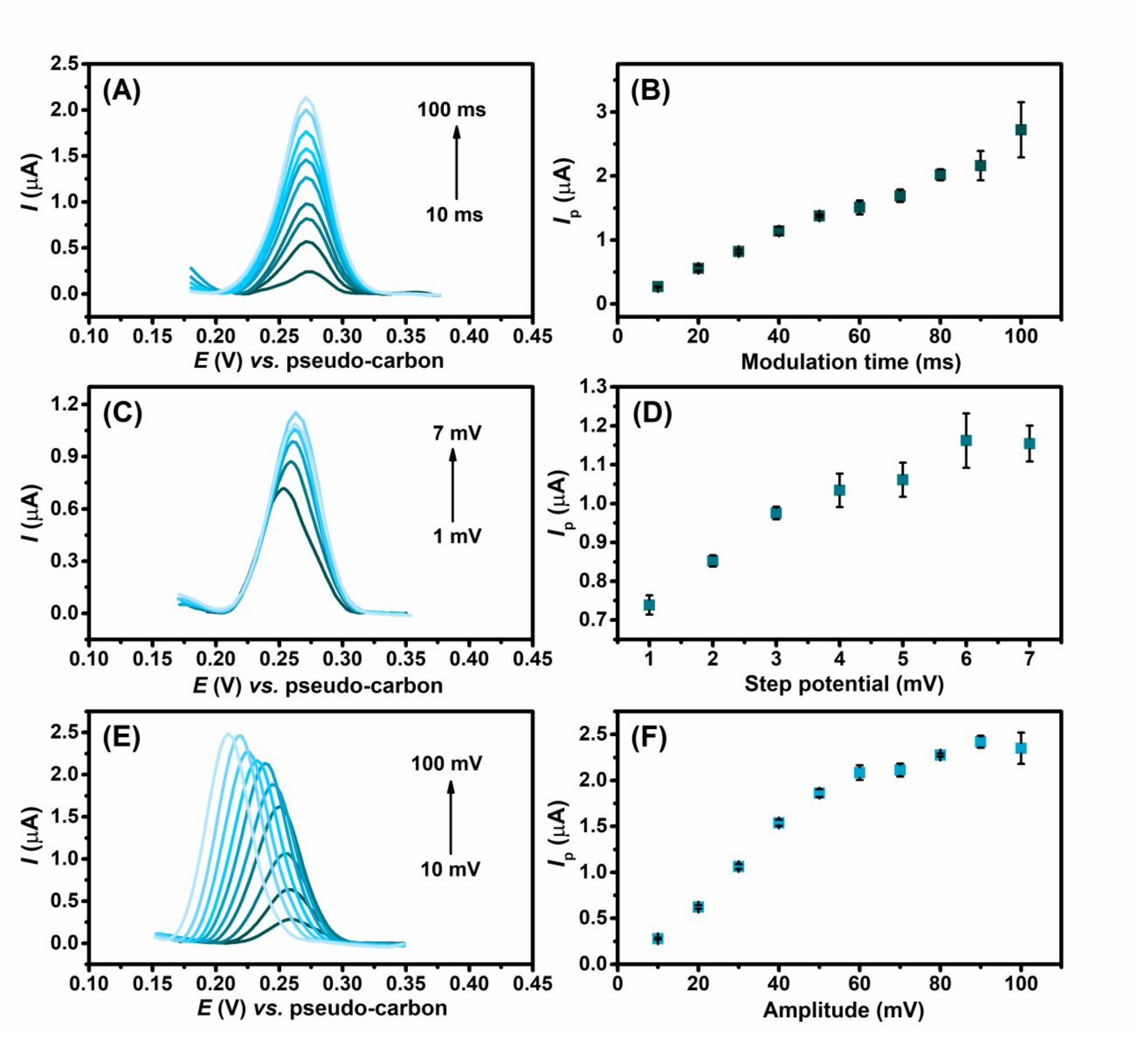

Figure S14: Baseline-corrected DPV responses recorded for 10 μmol L^{-1} UA under different experimental conditions: **(A)** modulation time (10–100 ms), **(C)** step potential (1–10 mV), and **(E)** pulse amplitude (10–100 mV). The corresponding plots of peak current versus parameter values are shown in **(B)**, **(D)**, and **(F)**, respectively. Supporting electrolyte: BR buffer (pH = 2.0).

Table S1: DPV parameters optimized for UA determination.

Parameter	Evaluated range	Optimized value

Modulation time (mV)	10 – 100	80
Step potencial (mV)	1 – 7	3
Amplitude (mV)	10 – 100	50

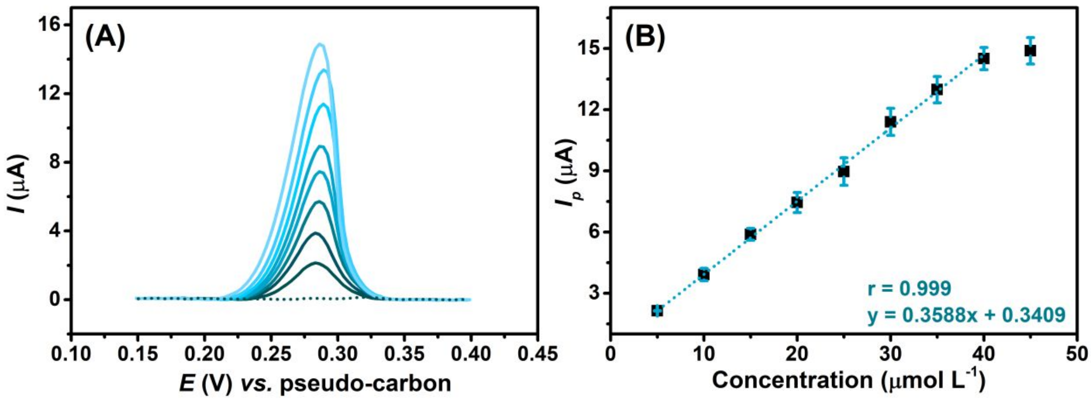

Figure S15: (A) Baseline-corrected DPV scans obtained with the PDE for increasing UA concentrations (5–45 µmol L⁻¹) and **(B)** corresponding calibration curve (mean of n = 3 replicates). **DPV conditions:** modulation time = 80 ms; pulse amplitude = 50 mV; step potential = 3 mV. Supporting electrolyte: BR buffer (pH = 2.0).

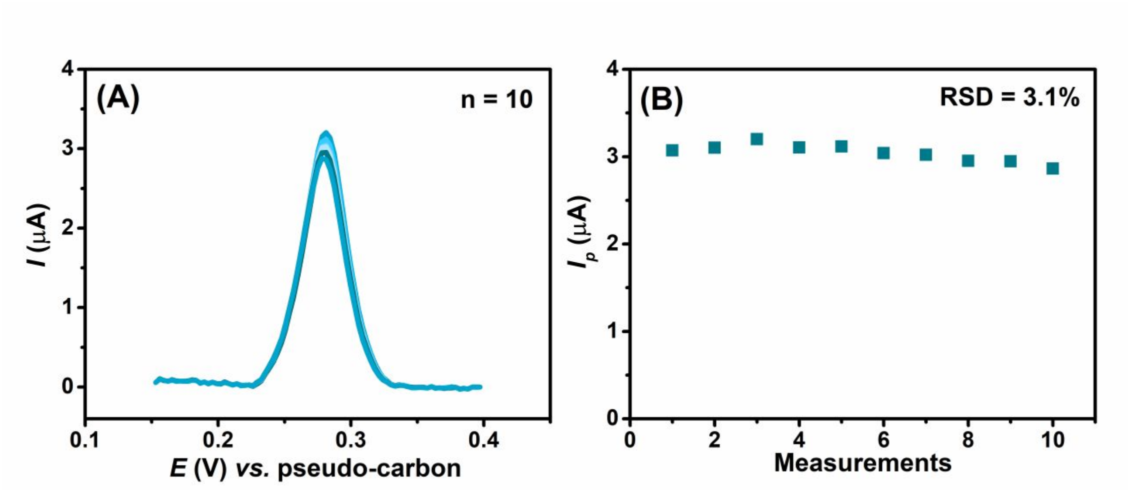

Figure S16: (A) Baseline-corrected DPV scans for 10 successive measurements of 10 µmol L⁻¹ UA at the PDE in BR buffer (pH 2.0). **(B)** Corresponding peak current (I_p) values for each measurement, with a relative standard deviation (RSD) of 3.1%. **DPV**

conditions: modulation time = 80 ms; pulse amplitude = 50 mV; step potential = 3 mV. Supporting electrolyte: BR buffer (pH = 2.0).

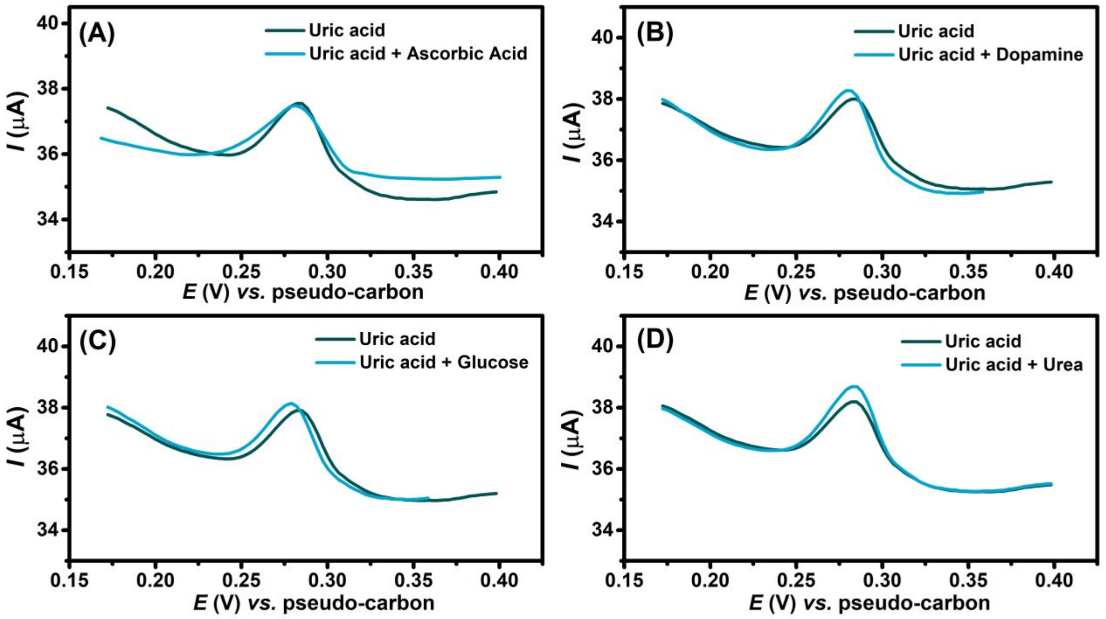

Figure S17: DPV obtained for uric acid in the absence and presence of potential interfering species: (A) ascorbic acid, (B) dopamine, (C) glucose, and (D) urea. Measurements were performed using 5 μmol L^{-1} of uric acid and 5 μmol L^{-1} of each interferent in BR buffer (pH 2.0). **DPV conditions:** modulation time = 80 ms; pulse amplitude = 50 mV; step potential = 3 mV.
